# Safety and efficacy of CAR T-cell therapy in central nervous system lymphoma: a systematic review and meta-analysis

**DOI:** 10.3389/fonc.2026.1790444

**Published:** 2026-03-23

**Authors:** Ahmad E. Shawabkeh, Jehad Yasin, Muaath I. Alsufi, Homam S. AbuHashesh, Hebah Khraisat, Zaid Muhanna, Izere Salomon, Salma A. Salman, Ahmad Obeid, Layan AlDaher

**Affiliations:** 1 School of Medicine, University of Jordan, Amman, Jordan; 2 College of Medicine and Health Sciences, University of Rwanda, Kigali, Rwanda; 3 School of Medicine, Ain Shams University, Cairo, Egypt

**Keywords:** CAR T-cell therapy, central nervous system lymphoma, efficacy, meta-analysis, neurotoxicity

## Abstract

**Background:**

The safety and efficacy of Chimeric Antigen Receptor (CAR) T-cell therapy in Central Nervous System Lymphoma (CNSL) remain uncertain, given the limited representation of CNS involvement in pivotal CAR T-cell trials. This meta-analysis synthesized data from studies evaluating outcomes in both primary (PCNSL) and secondary CNS lymphoma (SCNSL) cohorts treated with CART.

**Methods:**

A comprehensive search was performed across PubMed, Embase, and clinical registries up to October 2025. Studies reporting efficacy or toxicity outcomes in CNSL following CD19-targeted CART therapy were included. Proportions were stabilized using the Freeman-Tukey double arcsine transformation and pooled using inverse variance weighting. Between-study heterogeneity was estimated with the DerSimonian-Laird method, and τ² confidence intervals were calculated via the Jackson approach. Publication bias was assessed using Egger’s regression. Subgroup and multiple meta-regression analyses evaluated moderators including CNS category (PCNSL vs SCNSL) and publication year.

**Results:**

Data from thirty-eight studies were meta-analyzed. The pooled overall response rate (ORR) was 0.75 [95% CI: 0.70–0.79] with moderate-to-substantial heterogeneity (I² = 53.3%). Complete response (CR) rate was 0.52 [0.46–0.58], and partial response (PR) rate 0.18 [0.14–0.22]. No significant publication bias was detected (Egger’s p = 0.39 for ORR). Meta-regression indicated no significant effect of publication year or lymphoma subtype on response. Cytokine Release Syndrome (CRS) occurred in 83.5% [79.0–88.0], with grade ≥3 CRS in 5.77% [3.0–9.0]. Immune Effector Cell-Associated Neurotoxicity Syndrome (ICANS) was reported in 44.9% [36.0–54.0], with severe (grade ≥3) events in 17.4% [12.0–23.0]. Heterogeneity was substantial for ICANS (I² = 84.2%) but moderate for CRS (I² = 64.3%).

**Conclusions:**

CART therapy achieves robust response rates in CNS lymphoma, with efficacy comparable between PCNSL and SCNSL. Neurotoxicity remains frequent but manageable, and severe CRS events are infrequent.

**Systematic review registration:**

https://www.crd.york.ac.uk/prospero/, identifier CRD420251070033.

## 1 Introduction

Lymphoma of the central nervous system (CNS), both primary (PCNSL) and secondary (SCNSL), represents a rare but highly aggressive subset of non-Hodgkin lymphoma (NHL) (1). PCNSL is a rare variant of extra-nodal NHL that can impact sites anywhere along the entire neuroaxis, including the orbits, leptomeninges, brain, and spinal cord (1), with diffuse large B-cell lymphoma (DLBCL) being reported in 90% of cases (2). Secondary CNS lymphoma refers to systemic NHL that has disseminated to the CNS, which may occur as an isolated recurrence of previously diagnosed NHL or prevail simultaneously as a manifestation of systemic disease (1, 2).

Treatment of the central nervous system lymphomas (CNSL) typically involves intensive chemotherapy (commonly high-dose methotrexate-based regimens), followed by consolidation with autologous stem cell transplantation or whole-brain radiotherapy. However, for patients with relapsed or refractory disease, there is no established standard of care. Chimeric antigen receptor (CAR) T-cell therapy represents a paradigm shift in cancer immunotherapy, utilizing genetically modified autologous T lymphocytes engineered to recognize and eliminate malignant cells expressing specific tumor-associated antigens. The CAR construct comprises an extracellular single-chain variable fragment (scFv) that binds the target antigen, a transmembrane domain, and intracellular costimulatory domains (typically 4-1BB or CD28) coupled with CD3ζ signaling domain to activate T-cell cytotoxicity. Following ex vivo expansion and reinfusion, CAR T-cells undergo exponential *in vivo* proliferation upon antigen engagement, generating potent anti-tumor responses independent of major histocompatibility complex (MHC) restriction. Currently, four CD19-directed CAR T-cell products: Axicabtagene Ciloleucel (Yescarta^®^) (3), Tisagenlecleucel (Kymriah^®^) (4), Lisocabtagene Maraleucel (Breyanzi^®^) (5), and Brexucabtagene Autoleucel (Tecartus^®^) (6) have received regulatory approval for relapsed/refractory B-cell malignancies, demonstrating overall response rates of 52-82% and complete remission rates of 40-54% in pivotal trials (7, 8). The recent approval of anti-CD19 CAR T-cell therapy for systemic B-cell lymphomas has shifted the treatment landscape for previously incurable patients (9). Given that CD19 is nearly universally expressed in CNS lymphomas and that T cells are known to pass the blood brain barrier, there is a strong biological rationale for treating them with CAR T cells (10). CD19-directed CAR T-cells with costimulatory domains have shown high response rates and durable remissions in relapsed or refractory aggressive non-Hodgkin lymphoma, especially large B-cell lymphoma. Their use is now being explored in high-risk patients earlier in the treatment course, even before completion of first-line chemo-immunotherapy.

CD19-directed CAR T-cells have revolutionized treatment for relapsed/refractory B-cell malignancies, with four approved products demonstrating durable remissions (3–6). Early pivotal trials systematically excluded patients with active central nervous system (CNS) involvement primarily due to concerns over exacerbated neurotoxicity (e.g., cytokine release syndrome and immune effector cell-associated neurotoxicity syndrome), uncertain CAR T-cell trafficking across the blood-brain barrier, and lack of supportive preclinical safety data (11). This exclusion created a significant evidence gap, which our analysis aims to addresses (12). Several studies have recently shown promising efficacy of CD19-CAR T-cells in primary and secondary CNS lymphoma (13, 14). CAR T cells have since been shown to successfully traffic to the CNS, with modified T cells expressing CARs targeting a variety of primary brain tumor antigens demonstrating migration from the blood to tumor sites, and the efficiency of this migration is modifiable (14). Thus, this comprehensive review aims to synthesize current evidence on the role of CAR T-cell therapy in primary and secondary CNS lymphomas, with a focus on clinical outcomes, safety and tolerability profiles, and future therapeutic implications.

## 2 Methods

### 2.1 Protocol and registration

This systematic review and meta-analysis was conducted in accordance with the Preferred Reporting Items for Systematic Reviews and Meta-Analyses (PRISMA) 2020 guidelines (15). The review protocol was prospectively registered with PROSPERO CRD420251070033.

### 2.2 Search strategy and information sources

A comprehensive literature search was performed across multiple electronic databases, including PubMed/MEDLINE, Scopus, and Web of Science, from database inception through October 2025. The search strategy was developed in consultation with an experienced medical librarian and incorporated relevant Medical Subject Headings (MeSH) terms, Emtree terms, and free-text keywords. The following search concepts were combined using Boolean operators: (1) “chimeric antigen receptor” OR “CAR T-cell” OR “CAR-T” OR “CART”; (2) “central nervous system lymphoma” OR “CNS lymphoma” OR “primary CNS lymphoma” OR “PCNSL” OR “secondary CNS lymphoma” OR “SCNSL” OR “brain lymphoma” OR “leptomeningeal lymphoma”; and (3) “efficacy” OR “safety” OR “outcome” OR “response” OR “survival” OR “toxicity” OR “adverse events.” The complete search strategies for each database are provided in the **Supplementary Material** (**Supplementary Table 1**). Additionally, abstract databases from major hematology and oncology conferences, including the American Society of Clinical Oncology (ASCO), American Society of Hematology (ASH), and European Hematology Association (EHA), were manually searched from 2019 through 2025 to identify relevant conference abstracts and unpublished studies. Reference lists of included studies, relevant systematic reviews, and narrative reviews were hand-searched to identify additional eligible studies not captured by electronic database search. No language restrictions were applied during the initial search phase.

### 2.3 Eligibility criteria

Studies were included if they met the following criteria: (1) enrolled adult patients (≥18 years of age) diagnosed with either primary CNS lymphoma (PCNSL) or secondary CNS lymphoma (SCNSL), defined as systemic non-Hodgkin lymphoma with CNS involvement; (2) patients received CD19-based CAR T-cell constructs, including single-targeted (CD19, CD22, CD30) and dual/triple-targeted variants (CD19/CD22, CD19/CD20, CD20/CD22, CD19/CD20/CD22), including but not limited to Axicabtagene Ciloleucel, Tisagenlecleucel, or Lisocabtagene Maraleucel; (3) reported at least one efficacy outcome, including overall response rate (ORR), complete response (CR) rate, or partial response (PR) rate, assessed according to standardized response criteria (e.g., International Primary CNS Lymphoma Collaborative Group criteria or Lugano classification); or at least one safety outcome, including cytokine release syndrome (CRS) or immune effector cell-associated neurotoxicity syndrome (ICANS), graded according to consensus grading criteria (e.g., American Society for Transplantation and Cellular Therapy grading); and (4) published as full-text articles or conference abstracts in English.

Both prospective and retrospective study designs were eligible for inclusion, including randomized controlled trials, phase I and phase II clinical trials, prospective and retrospective cohort studies, and case series with three or more patients. Studies were excluded if they were (1) case reports with fewer than three patients, (2) review articles, editorials, commentaries, or letters to the editor without original data, (3) preclinical studies or *in vitro*/*in vivo* animal studies, or (4) studies that did not specify CNS involvement status or did not report outcomes separately for patients with CNS lymphoma. No restrictions were placed on prior lines of therapy, bridging therapy regimens, or geographic location of the study.

### 2.4 Study selection

Following the removal of duplicate records using Rayyan software and manual verification, two independent reviewers screened the titles and abstracts of all retrieved records for potential eligibility. Studies deemed potentially relevant by either reviewer advanced to full-text review. The same two reviewers then independently assessed the full-text articles against the predefined eligibility criteria. Discrepancies between reviewers at both screening stages were resolved through discussion and consensus. When consensus could not be reached, a third senior reviewer was consulted to make the final decision. When multiple publications reported data from overlapping patients, the most recent or comprehensive report was included to avoid duplication of patients’ data. Authors of studies with insufficient or unclear data were contacted via email to request additional information or clarification.

### 2.5 Data extraction

Data extraction was performed independently by two reviewers using a standardized, pilot-tested data extraction form developed in Microsoft Excel. The following information was extracted from each included study: (1) study characteristics: first author name, year of publication, country or countries of origin, study design (prospective vs. retrospective, phase of clinical trial if applicable), sample size, and duration of follow-up; (2) patient demographics and disease characteristics: median or mean age, sex distribution, CNS lymphoma classification (PCNSL, SCNSL, or both), histologic subtype (e.g., diffuse large B-cell lymphoma), sites of CNS involvement (e.g., parenchymal, leptomeningeal, intraocular), and number of prior lines of therapy; (3) CAR T-cell product characteristics: specific CAR T-cell product (axicabtagene ciloleucel, tisagenlecleucel, lisocabtagene maraleucel, or other), and CAR T-cell dose; (4) treatment details: bridging therapy administered (yes/no and type if reported), and conditioning chemotherapy regimen; (5) efficacy outcomes: number of patients achieving overall response (complete response plus partial response), complete response, and partial response, overall survival (OS) and progression-free survival (PFS) rates at different timepoints, hazard ratios (HRs), median OS (mOS), and median PFS (mPFS); and (6) safety outcomes: number of patients experiencing any-grade CRS and grade ≥3 CRS, and any-grade ICANS and grade ≥3 ICANS, graded according to the American Society for Transplantation and Cellular Therapy (ASTCT) consensus grading criteria or equivalent.

For studies that reported outcomes at multiple time points, data from the time point with the longest follow-up were extracted. When outcomes were reported separately for PCNSL and SCNSL subgroups within a single study, data for each subgroup was extracted separately. Discrepancies in data extraction were identified through comparison and resolved through discussion with a third reviewer if necessary.

### 2.6 Quality assessment

The methodological quality and risk of bias of included studies were assessed independently by two reviewers using validated quality assessment tools appropriate for the specific study designs.

For single-arm retrospective cohort studies (n=28), a modified version of the Newcastle-Ottawa Scale (NOS) was utilized (16). Given the single-arm nature of these studies, the standard “Comparability” domain was excluded. This modified scale evaluated studies across two domains with a maximum possible total score of 6 stars, the quality of the included cohort studies was assessed using the Newcastle-Ottawa Scale (NOS). Under the Selection domain (maximum of three stars), one star was assigned for the representativeness of the exposed cohort, one for the ascertainment of exposure, and one for the demonstration that the outcome of interest was not present at the start of the study. Under the Outcome domain (maximum of three stars), one star was awarded for the assessment of outcome, one for adequate follow-up duration to allow outcomes to occur, and one for the adequacy of cohort follow-up.

One star was awarded for each satisfied criterion. Follow-up duration was deemed adequate if it allowed for the assessment of acute toxicities and initial responses (typically ≥1 month post-infusion), as most events occur early after CAR T-cell therapy. Based on this modified scale, studies with a total score of 5–6 stars were classified as good quality, while studies with a score of ≤4 stars were classified as moderate quality. The detailed NOS assessments for all cohort studies are provided in **Supplementary Table 3**.

For single-arm interventional studies (n=11), including phase I and phase II clinical trials and prospective case series, the Methodological Index for Non-Randomized Studies (MINORS) was applied (17). MINORS consists of 8 items. Each item was scored as 0 (not reported), 1 (reported but inadequate), or 2 (reported and adequate), with a maximum possible score of 16 for non-comparative studies. The following domains were assessed: (1) a clearly stated aim, (2) inclusion of consecutive patients, (3) prospective collection of data, (4) endpoints appropriate to the aim of the study, (5) unbiased assessment of the study endpoint, (6) follow-up period appropriate to the aim of the study, (7) loss to follow-up less than 5%, and (8) prospective calculation of the study size. Studies with scores ≥12 (of 16) were considered good quality, and studies scoring 10–11 were classified as moderate quality. The detailed MINORS assessments for all interventional studies are provided in **Supplementary Table 4**.

Quality assessment focused on the following key methodological aspects: clarity of study aims and objectives, clarity of inclusion and exclusion criteria, prospective or retrospective data collection, appropriate outcome assessment methods, adequate follow-up duration, reporting of loss to follow-up, and appropriate statistical analysis. Disagreements in quality assessment were resolved by consensus or by consulting a third senior reviewer. Studies were not excluded based on quality assessment scores; however, quality scores were considered in sensitivity analyses and in the interpretation of results.

### 2.7 Statistical analysis

All statistical analyses were conducted using R (R Foundation for Statistical Computing, Vienna, Austria) (18), primarily employing the ‘meta’ package (19). Proportions were pooled using the inverse-variance method with the Freeman–Tukey double arcsine transformation to stabilize variances, particularly in studies reporting rare events. For outcomes with zero events in one study arm, a continuity correction of 0.5 was applied, while studies with zero total events were excluded from the analysis. For the evaluation of survival outcomes, the weighted median of medians was calculated to estimate the aggregated mOS and mPFS across the study cohorts (20). Between-study heterogeneity was assessed using Cochran’s Q statistic, with corresponding I² values calculated to quantify the proportion of variability attributable to heterogeneity rather than sampling error. The DerSimonian–Laird (DL) estimator was used to estimate the between-study variance (τ²), and Jackson’s method was applied to derive confidence intervals for τ² and τ. In accordance with predefined criteria, a random-effects model was used when heterogeneity was substantial (I² ≥ 50%), whereas a fixed-effect model was applied when heterogeneity was low (I² < 50%). Prediction intervals were calculated based on a t-distribution with degrees of freedom equal to k − 1 (df = 38), providing an estimate of the expected range of effects in future studies.

To explore potential sources of heterogeneity, subgroup analyses were performed based on clinical group classification. In addition, a mixed-effects multiple meta-regression was conducted to assess the influence of predefined moderators, including publication year, study group, and study design (observational vs interventional), while accounting for residual heterogeneity across studies.

Robustness of pooled estimates was evaluated using leave-one-out (LOO) sensitivity analyses, whereby each study was sequentially omitted to assess its influence on the overall effect size. Publication bias was examined visually using funnel plots and statistically using Egger’s linear regression test, with a two-sided p-value < 0.05 considered indicative of small-study effects. All statistical tests were two-sided, and results are reported with corresponding 95% confidence intervals unless otherwise stated.

## 3 Results

### 3.1 Search results

**Figure 1:**
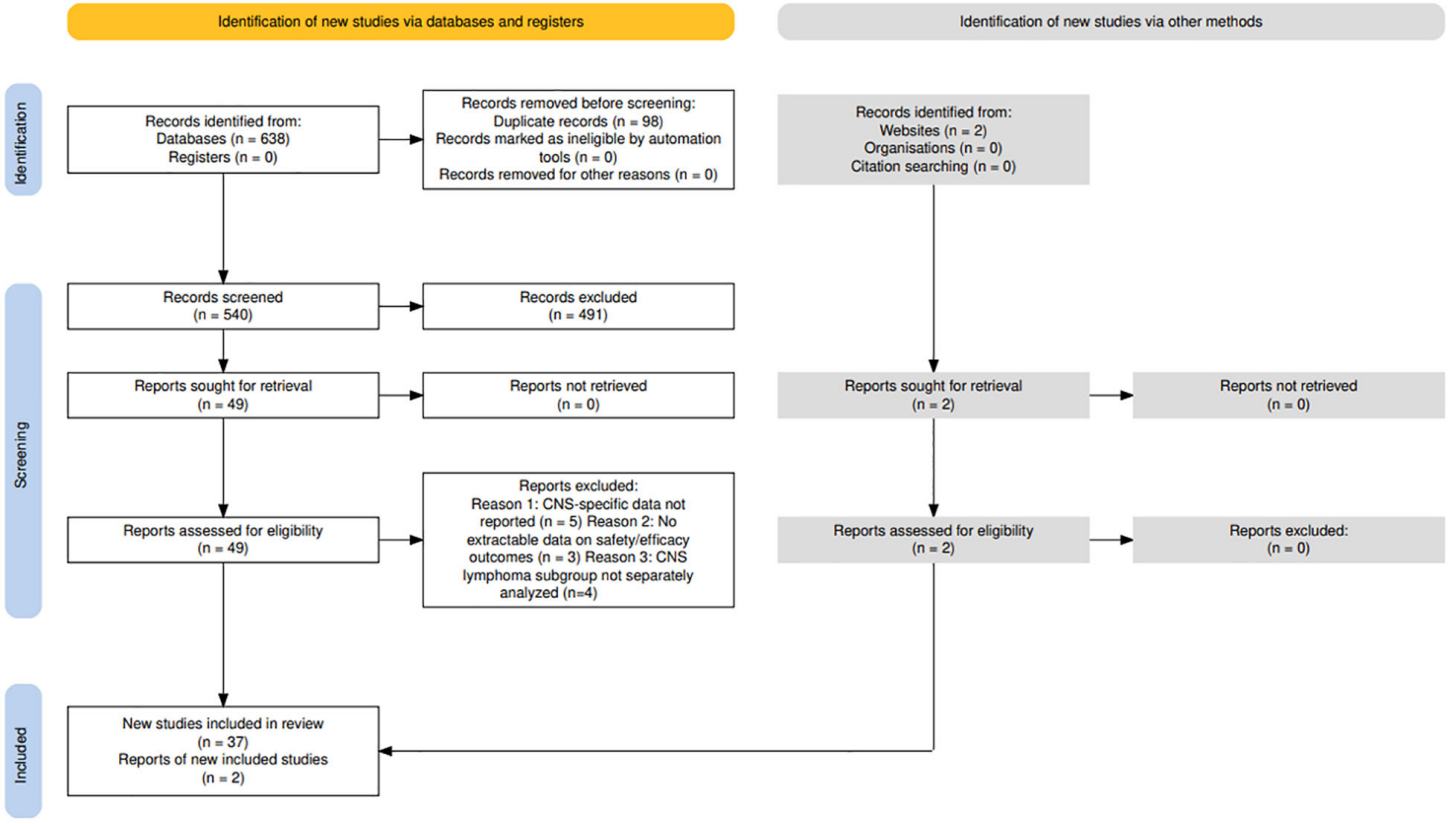
Flowchart illustrating the identification, screening, eligibility assessment, and inclusion of studies in the systematic review and meta-analysis. A total of 638 records were identified through database searches, with 98 duplicates removed. After title/abstract screening, 49 reports were sought for retrieval and 49 were assessed for eligibility. Studies were excluded primarily due to CAR-T–specific data not being reported or insufficient extractable safety/efficacy outcomes. Two additional records were identified through other methods. Ultimately, 39 studies met the inclusion criteria and were included in the final qualitative and quantitative synthesis.

Our initial search identified 638 studies across (PubMed, Scopus, and Web of Science). After removing duplicates, 540 studies underwent title and abstract screening, resulting in the exclusion of 491 studies. The remaining 49 full-text studies were independently reviewed by the same investigators. Of these, 37 were deemed eligible for inclusion. 2 additional studies were identified from 2 websites (American Society of Clinical Oncology) and subsequently included. Meta-analysis was conducted on 39 of these studies. The study selection process is illustrated in the PRISMA flowchart in **Figure 1**.

### 3.2 Characteristics of included studies

Our study included data from 39 studies. 38 studies were included in quantitative analysis, with a maximum sample size of 1,190 patients in our analyses. Most studies were retrospective cohorts, with the exception of 7 studies (8, 21–26) that were phase I and phase II clinical trials, and a prospective case series. The studies were conducted in the USA (n=22) (7, 7, 8, 13, 13, 21, 23–25, 27–38), China (n = 10) (22, 26, 39–46), France (n = 3) (10, 47, 48), Germany (n = 2) (49, 50), Switzerland (n = 1) (51), EU (n = 1) (52). One study did not mention the country (31). Among the studies, 20 studies evaluated SCNSL (7, 8, 13, 22, 24, 27–33, 35, 37, 39, 45, 49, 50, 53), 5 evaluated PCNSL (21, 25, 36, 47, 48), and 13 included both (10, 13, 23, 26, 34, 38, 40–44, 51, 52). The basic characteristics of the included studies are shown in **Table 1** and **Table 2**.

**Table 1:** Overview of study design, geographic setting, follow-up duration, and the spectrum of reported safety and efficacy endpoints across 32 studies (2019–2025) evaluating CAR T-cell therapy in primary (PCNSL) or secondary (SCNSL) CNS lymphoma.

Author	Year of publication	Type of study	Location of study	Median follow up (months)	Reported safety outcomes	Reported efficacy outcomes
Kline et al. (35)	2024	Prospective case series	USA	2.9	ICANS any grade; ICANS ≥3; HLH any grade	ORR, CR, PR, PFS, OS
Wu et al.	2025	Retrospective cohort	China	37.5	CRS any grade; CRS ≥3; ICANS any grade; ICANS ≥3; cytopenias ≥3; infections ≥3	ORR, CR, PR, PFS, OS, DOR, OS rate, PFS rate
Shi et al. (41)	2025	Retrospective cohort	China	12	CRS any grade; ICANS any grade; ICANS ≥3	ORR, CR, PR, PFS, HR-PFS (PCNSL vs SCNSL), OS, HR-OS (PCNSL vs SCNSL), OS rate, PFS rate
Epperla et al. (31)	2023	Retrospective cohort	USA	14.1	CRS any grade; CRS ≥3; ICANS any grade; ICANS ≥3	ORR, CR, PR, PFS, OS, DOR, OS rate, PFS rate
Zhou et al. (46)	2024	Letter to Editor/Retrospective cohort	China	16.73	CRS any grade; CRS ≥3; ICANS any grade; cytopenias ≥3; infections ≥3	ORR, CR, PR, PFS, OS, OS rate, PFS rate, HR-PFS (PCNSL vs SCNSL)
Mercadal et al. (36)	2025	Letter to Editor/Retrospective cohort	USA	26	CRS any grade; ICANS any grade; ICANS ≥3	ORR, CR, PR, PFS, OS, OS rate, PFS rate
Lacan et al. (10)	2023	Letter to Editor/Retrospective cohort	France	12	CRS any grade; CRS ≥3; ICANS any grade; ICANS ≥3	ORR, CR, PR, PFS, OS, OS rate, PFS rate
Alsouqi et al.	2023	Letter to Editor/Retrospective cohort	USA	10.7	CRS any grade; CRS ≥3; ICANS any grade; ICANS ≥3	ORR, CR, PR, PFS, OS, OS rate, PFS rate
Choquet et al.	2023	Retrospective cohort	France	20.8	CRS any grade; CRS ≥3; ICANS any grade; ICANS ≥3; cytopenias ≥3; neurotoxicity any grade	ORR, CR, PR, PFS, OS, OS rate, PFS rate, HR-OS (CART vs Control)
Frigault et al. (32)	2019	Retrospective cohort	USA	NR	CRS any grade	ORR, CR, PR
Alcantara et al. (47)	2022	Retrospective cohort	France	8.5	CRS any grade; CRS ≥3; ICANS any grade; ICANS ≥3; cytopenias ≥3 (prolonged)	ORR, CR, PR, PFS, OS
Frigault et al. (21)	2022	Prospective phase 1/2	USA	12.2	CRS any grade; ICANS any grade; ICANS ≥3	ORR, CR, PR, PFS, OS
Epperla et al. (53)	2024	Retrospective Cohort	N/A	2	CRS any grade; CRS ≥3; ICANS any grade; ICANS ≥3	ORR, CR, PR, PFS, OS, OS rate, PFS rate
Lin et al. (39)	2024	Retrospective cohort	China	NR	CRS any grade; CRS ≥3; ICANS any grade; ICANS ≥3; cytopenias ≥3; infections ≥3	NR
Zhang et al. (45)	2022	Retrospective cohort	China	12	CRS any grade; ICANS any grade; ICANS ≥3; cytopenias ≥3; organ toxicity (GI, liver, renal, cardiac) any grade	ORR, CR, PR, PFS, OS, OS rate, PFS rate
Saidy et al. (52)	2025	Retrospective cohort	EU	24.8	CRS any grade; CRS ≥3; ICANS any grade; ICANS ≥3; cytopenias any grade; infections ≥3; deaths from infection	ORR, CR, PR, PFS, OS
Shumilov et al. (51)	2023	Retrospective cohort	Switzerland	4.9	CRS any grade; CRS ≥3; ICANS any grade; ICANS ≥3; HLH any grade; cytopenias any grade; infections any grade; deaths from infection	ORR, CR, PR, PFS, OS
Yu et al. (44)	2024	Retrospective cohort	China	10.5	CRS any grade; CRS ≥3; ICANS any grade; ICANS ≥3; cytopenias any grade; infections any grade; hepatic dysfunction any grade	ORR, CR, PR, OS rate
Liu et al. (40)	2022	Prospective Clinical Trial, Single-Arm, Single-Center (Nonrandomized)	China	10.4	CRS any grade; ICANS any grade; cytopenias any grade	ORR, CR, PR
Riedell et al.	2025	Retrospective cohort	USA	15.5	ICANS any grade; ICANS ≥3	ORR, CR, PR
Karschnia et al. (34)	2023	Retrospective cohort	USA	12.0	CRS any grade; CRS ≥3; ICANS any grade; ICANS ≥3	ORR, CR, PR, PFS, OS
Wang et al.	2023	Phase I, open-label, multicenter, seamless design	USA	16.1	CRS any grade; CRS ≥3; ICANS any grade; ICANS ≥3; cytopenias ≥3; infections ≥3; hypogammaglobulinemia any grade	ORR, CR, PR, PFS, OS, OS rate, PFS rate
Epperla et al.	2025	Multicenter retrospective real-world cohort study	USA	48.2	CRS any grade; CRS ≥3; ICANS any grade; ICANS ≥3	ORR, CR, PR, PFS, OS, OS rate, PFS rate
Nayak et al. (23)	2024	Pilot study, single-arm, phase I/II style (safety & efficacy endpoints	USA	24.2	CRS any grade; ICANS any grade; ICANS ≥3	ORR, CR, PR, PFS, OS, OS rate, PFS rate
Wu et al. (26)	2021	Single-center, open-label, single-arm clinical trial	China	14.2	CRS any grade; ICANS any grade; ICANS ≥3; infections any grade	ORR, CR, PR, OS rate, PFS rate
Ghafouri et al. (33)	2021	Retrospective, single-institution case series	USA	11.5	CRS any grade; ICANS any grade; ICANS ≥3; infections any grade	ORR, CR, PR, PFS, OS
Sanber et al. (38)	2023	Retrospective cohort	USA	13.8	CRS any grade; ICANS any grade	ORR, CR, PR
Ryan et al. (37)	2023	Retrospective cohort	USA	15.4	CRS any grade; CRS ≥3; ICANS any grade; ICANS ≥3	ORR, CR, PR, OS rate, PFS rate
Ahmed et al. (28)	2024	Retrospective cohort	USA	16.7	NR	ORR, CR, PR, OS rate, PFS rate
Ahmed et al. (27)	2021	Retrospective cohort	USA	5.1	CRS any grade; CRS ≥3; ICANS any grade; ICANS ≥3	ORR, CR, PR, PFS, OS
Siddiqi et al. (25)	2021	Phase 1 clinical trial	USA	NR	CRS any grade; CRS ≥3	ORR, CR, PR, OS

Predominantly retrospective cohorts from the USA, China, Europe, and France were included. Median follow-up ranged from 2 to 48 months (about 4 years); safety outcomes focused on CRS/ICANS (any/≥grade 3), cytopenias, infections, and HLH. Efficacy endpoints encompassed ORR, CR/PR, and PFS/OS (with HRs/rates/DOR reported). CR, complete response; CRS, cytokine release syndrome; DOR, duration of response; HR, hazard ratio; HR-OS, hazard ratio for overall survival; HR-PFS, hazard ratio for progression-free survival; ICANS, immune effector cell-associated neurotoxicity syndrome; HLH, hemophagocytic lymphohistiocytosis; NR, not reported; ORR, overall response rate; OS, overall survival; PFS, progression-free survival; PR, partial response.

**Table 2:** Demographic and clinical characteristics of patients treated with CAR T-Cell therapy for CNS lymphoma.

Author	Year of publication	Population	Analyzed/total number of patients	Median age	M:F	Tumor histology	CAR-T cell product	Prior ASCT n(%)
Kline et al. (35)	2024	SCNSL	4/4	38.3	4:0	Burkitt (2/4) Non-GCB: DLBCL (1/4), HGBCL (1/4)	Axi-cel, Tisa-cel, Liso-cel	1 (25)
Wu et al.	2025	Both	38/38	47	23:15	DLBCL (36/38), Burkitt (1/38), Intravascular large B-cell Lymphoma (1/38)	Axi-cel, Relma-cel	2 (5.3)
Shi et al. (41)	2025	Both	27/27	58	15:12	PCNSL (19/27), SCNSL (8/27); DLBCL (5/27), PMBL (1/27), BL (1/27), Richter’s (1/27).	CD19 Directed CD20 Directed CD19/CD22 Directed	6 (22.2)
Epperla et al. (31)	2023	SCNSL	61/61	56	34:27	*De novo* DLBCL (48/61) Transformed Lymphoma (7/61), Others (4/61)	Axi-cel, Tisa-cel, Liso-cel, Brexu-cel	14 (23)
Zhou et al. (46)	2024	Both	ASCT + CART (29/29) CART (10/10) CIT (17/17)	42 38.5 62	14:15 3:7 10:7	DLBCL (29/29) DLBCL (10/10) DLBCL (17/17)	CD19/CD22 Directed	25 (100), 0 (0)
Mercadal et al. (36)	2025	PCNSL	24/24	57	16:8	DLBCL (23/23)	Axi-cel, Tisa-cel	12 (50)
Lacan et al. (10)	2023	Both	21/21	67	11:10	NR	Axi-cel, Tisa-cel	16 (76.2)
Alsouqi et al.	2023	SCNSL	80/86	62	53:33	DLBCL (67/86), tFL (12/86), HGBCL (6/86), Burkitt lymphoma (1/86)	Axi-cel, Tisa-cel, Liso-cel	22 (19)
Choquet et al.	2023	PCNSL	27/247	68	14:13	DLBCL (27/27)	Axi-cel, Tisa-cel	14 (52)
Matthew J. Frigault (32)	2019	SCNSL	8/8	50	4:4	DLBCL (5/8), PMBCL(1/8), HGBCL(2/8)	Tisa-cel	1 (12.5)
Marion Alcantara (47)	2022	PCNSL	9/9	67	3:6	NR	Axi-cel, Tisa-cel	7 (77.8)
Matthew J. Frigault (21)	2022	PCNSL	12/12	63	7:5	LBCL (12/12)	Tisa-cel	3 (25)
Narendranath Epperla (53)	2024	SCNSL	136/144	61	52:92	NR	Axi-cel, Tisa-cel, Liso-cel	44 (31)
Haolong Lin et al. (39)	2024	SCNSL	ASCT+CART: 23/26 CART: 3/26	NR	NR	BCL (26/26)	CD19 Directed CD22 Directed CD30 Directed CD19/22 Directed CD19/20 Directed CD20/22 Directed CD19/20/22 Directed	23 (88.5)
Huanxin Zhang et al. (45)	2022	SCNSL	15/15	51	11:4	DLBCL (13/15), PMBCL (1/15), Burkitt (1/15)	CD19 Directed CD19/20 Directed CD19/22 Directed	2 (10.5)
Anna Ossami Saidy et al. (52)	2025	Both	100/106	62	62:38	DLBCL (86/100) High‐grade lymphoma (Myc + Bcl‐2 ± Bcl‐6) (6/100) Others (8/100)	Axi-cel, Tisa-cel, Brexu-cel	40 (40)
Evgenii Shumilov et al. (51)	2023	Both	15/15	61	8:7	DLBCL (15/15) *De novo* (13/15)	Axi-cel, Tisa-cel	9 (60)
Wenyan Yu (44)	2024	Both	22/22	56	14:8	DLBCL GCB 13 (59.1) DLBCL non-GCB 9 (40.9) DHL/THL 4 (18.2)	Relma-cel	1 (4.5)
Rui Liu (40)	2022	Both	7/7	48	3:4	DLBCL	CD19 Directed CD20 Directed	2 (28.6)
Peter A. Riedell	2025	SCNSL	10/101	NR	NR	NR	Liso-cel	NR
Philipp Karschnia (34)	2023	Both	45/45	32	26:19	DLBCL (37/45) Burkitt (1/45) Transformed lymphoma (9/45)	Axi-cel, Tisa-cel, Liso-cel	9 (20.0)
Michael Wang	2023	SCNSL	7/88	68.5	67:21	Mantle Cell Lymphoma	Liso-cel	29 (33)
Narendranath Epperla	2025	SCNSL	65/65	63	43:22	DLBCL (all included); subset with double-/triple-hit lymphoma (12/50)	Axi-cel	12(18)
Lakshmi Nayak (23)	2024	Both	17/18	62	10:8	DLBCL	Tisa-cel	NR
Jiaying Wu (26)	2021	Both	4/13	42	6:7	All DLBCL including 4 Primary CNSL, 9 Secondary CNSL	BrexU-cel	0
Sanaz Ghafouri (33)	2021	SCNSL	5/5	63	3:2	All DLBCL	Axi-cel	1 (20)
Khaled Sanber (38)	2023	Both	3/3	45	2:1	DLBCL (1/3), B-ALL (Ph-) 1/3, NS 1/3	Axi-cel, Brexu-cel	1 (33.3)
Christine E. Ryan (37)	2023	SCNSL	7/7	60.1	4:3	MCL (7/7): Blastoid (3/7), Classic (1/7), Ploeomorphic (1/7), NS (2/7)	Tisa-cel, Brexu-cel	2 (28.6)
Gulrayz Ahmed (28)	2024	SCNSL	8/8	72	1:7	Mantle cell lymphoma (MCL)	Brexu-cel	NR
Gulrayz Ahmed (27)	2021	SCNSL	7/7	50	4:3	DLBCL	Axi-cel, Tisa-cel	NR
Tanya Siddiqi (25)	2021	PCNSL	5/5	49	0:5	DLBCL	CD19 Directed	NR
Fei Xue (43)	2022	Both	17/17	42	9:8	DLBCL (15/17), MCL (1/17), BL (1/17)	4-1BB Based Targeting CD19/CD20/CD22	3 (17.6)
Francis Ayuk (49)	2023	SCNSL	28/28	58	16:12	N/A	Axi-cel, Tisa-cel	NR
Philipp Karschnia (50)	2022	SCNSL	10/10	55	6:4	DLBCL (6/10), trFL (3/10), PTLD (1/10)	CD19 Directed	NR
Jae H. Park (24)	2023	SCNSL	5/5	64	2:3	DLBCL (2/5), HGBCL (2/5), MCL (1/5)	Axi-cel, Tisa-cel, Brexu-cel	3(60)
Jeremy S Abramson (7)	2020	SCNSL	6/7	NR	NR	NR	Liso-cel	NR
He et al. (22)	2025	SCNSL	21/21	NR	9:12	B-ALL (11/21), B-NHL (10/21): DLBCL (7), Burkitt (1), HGBL (1), B-LBL (1)	1928zT2 cells (3^rd^ Generation CAR-T)	8 (38)
Hernández-Tost et al.	2025	Both	48/48	62	27:21	DLBCL (46/48), Mantle Cell Lymphoma (2/48)	Axi-cel, Tisa-cel, Liso-cel	26 (54)
Bennani et al. (13)	2019	SCNSL	15/17	58	11:6	B-cell NHL	Axi-cel	7 (41)

The summarizes baseline characteristics from 39 studies (2019-2025) treated with CAR T-cell therapy for primary or secondary CNS lymphoma. Data extracted include patient demographics (age, sex), CNS lymphoma type, tumor histology, CAR T-cell product administered, and prior autologous stem cell transplantation (ASCT) exposure. Diffuse large B-cell lymphoma (DLBCL) was the predominant histology. Median age ranged from 32–72 years, and prior ASCT exposure varied from 0-100%, reflecting heterogeneous patient populations across studies. ASCT, autologous stem cell transplantation; CAR T, chimeric antigen receptor T-cell; DLBCL, diffuse large B-cell lymphoma; NR, not reported; PCNSL, primary CNS lymphoma; SCNSL, secondary CNS lymphoma. BL, Burkitt lymphoma; B-ALL, B-cell acute lymphoblastic leukemia; B-NHL, B-cell non-Hodgkin lymphoma; B-LBL, B-lymphoblastic lymphoma; BCL, B-cell lymphoma; CNS, Central nervous system; DLBCL, Diffuse large B-cell lymphoma; GCB, Germinal center B-cell subtype; HGBCL/HGBL, High-grade B-cell lymphoma; LBCL, Large B-cell lymphoma; MCL, Mantle cell lymphoma; NR, Not reported; NS, Not specified; PCNSL, Primary central nervous system lymphoma; Ph-, Philadelphia chromosome negative; PMBCL/PMBL, Primary mediastinal B-cell lymphoma; PTLD, Post-transplant lymphoproliferative disorder; SCNSL, Secondary central nervous system lymphoma; tFL/trFL, Transformed follicular lymphoma; DHL, Double-hit lymphoma; THL, Triple-hit lymphoma; Myc, MYC gene rearrangement.

### 3.3 Quality assessment

Risk of bias was evaluated using modified NOS (16), and MINORS (17) quality assessment tools for the 39 included studies. 33 studies were considered of good quality and 6 were of moderate quality. Many studies did not report data on loss to follow-up. However, overall assessment revealed that most studies had good quality. The details of the quality assessment are summarized in the **Supplementary Material** (**Supplementary Tables 3**, **4**).

### 3.4 Efficacy outcomes

#### 3.4.1 Survival outcomes

Survival outcomes are summarized in **Supplementary Figures** (**Supplementary Figures 1**, **3**). The pooled hazard ratio for progression-free survival (PFS) comparing PCNSL versus SCNSL showed no significant difference between groups (random-effects HR 0.89, 95% CI: 0.16–4.91) (**Supplementary Figure 1**). Time-stratified analyses demonstrated OS rates from 0.71 (95% CI 0.41–0.94) at 6 months to 0.48 (95% CI: 0.31–0.66) at 24 months (**Supplementary Figure 2**). Similarly, PFS rates decreased with follow-up time, from 0.41 (95% CI: 0.22–0.61) at 6 months to 0.28 (95% CI 0.20–0.37) at 24 months.

Across studies evaluating CAR T-cell therapy in CNS lymphoma, reported median overall survival (mOS) ranged from 4.3 to 26.4 months, while median progression-free survival (mPFS) ranged from 2 to 16.3 months. The pooled median of medians was 8.6 months (95% CI: 7.6–15.0) for mOS and 3.6 months (95% CI: 3.0–4.72) for mPFS (**Supplementary Table 5**).

#### 3.4.2 Overall response rate

**Figure 2:**
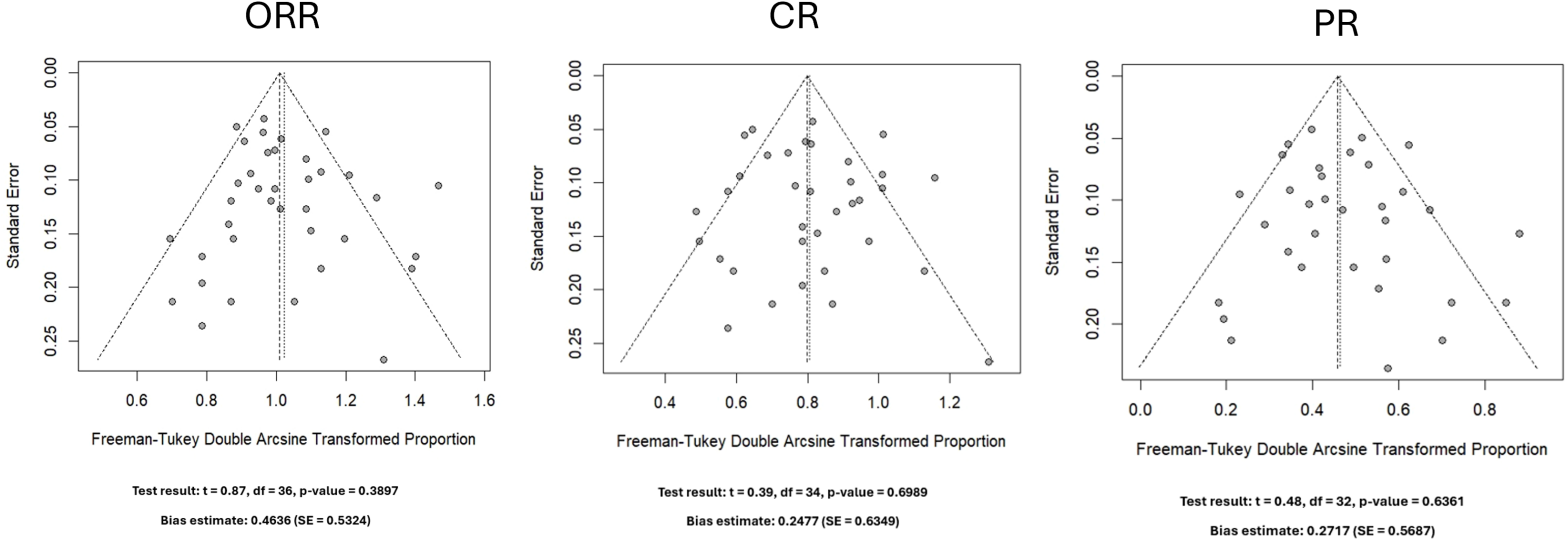
Forest plots showing pooled estimates for ORR, CR, and PR. ORR, Objective Response Rate; CR, Complete Response; PR, Partial Response.

**Figure 3:**
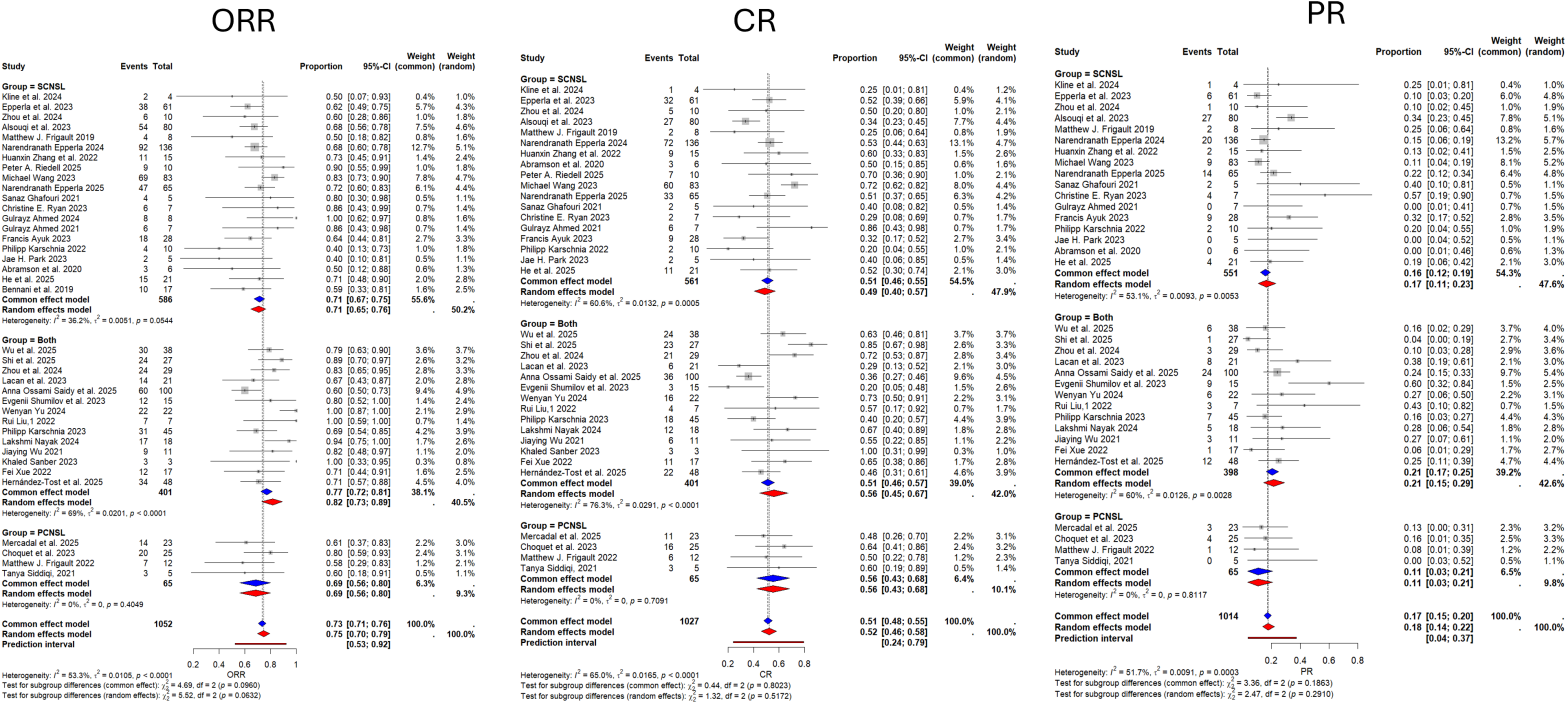
Funnel plots and Egger’s regression test results for ORR, CR, and PR estimates across meta-analyzed studies. ORR, Objective Response Rate; CR, Complete Response; PR, Partial Response.

A total of 1052 participants across 38 studies were included in the ORR analysis. The pooled ORR across all studies was 0.75 (95% CI: 0.70-0.79), with a prediction interval of 0.53 to 0.92. Moderate-to-substantial heterogeneity was observed among the included studies (I^2^ = 53.3%, τ^2^ = 0.0105, p < 0.0001). Subgroup analysis stratified by CNS lymphoma classification showed no significant difference in ORR between PCNSL and SCNSL (p = 0.0632), with a pooled HR of 0.71 (95% CI: 0.65–0.76) for SCNSL, 0.69 (95% CI: 0.56–0.80) for PCNSL, and 0.82 (95% CI: 0.73–0.89) for studies including both. The forest plot for ORR and subgroup estimates is shown in **Figure 2**. No evidence of publication bias was detected using Egger’s test (p = 0.39) (**Figure 3**). ORR stratified by CAR-T product are presented in **Supplementary Figure 4**. Product-specific random-effects estimates were 0.72 (95% CI: 0.67–0.76) for multi-product studies, 0.72 (95% CI: 0.37–0.97) for tisagenlecleucel, 0.82 (95% CI: 0.64–0.95) for lisocabtagene maraleucel, 0.71 (95% CI: 0.61–0.81) for axicabtagene ciloleucel, and 0.93 (95% CI: 0.67–1.00) for brexucabtagene autoleucel. Subgroup differences were statistically significant under the random-effects model (p = 0.0004).

#### 3.4.3 Complete response

A total of 1,027 participants across 36 studies were included in the CR analysis. The pooled CR rate across all studies was 0.52 (95% CI: 0.46–0.58), with a prediction interval of 0.24 to 0.79. Substantial heterogeneity was observed among the included studies (I^2^ = 65.0%, τ^2^ = 0.0165, p < 0.0001). Subgroup analysis stratified by CNS lymphoma classification showed no significant difference in HR for CR between PCNSL and SCNSL (p = 0.5172), with a pooled HR of 0.49 (95% CI: 0.40–0.57) for SCNSL, 0.56 (95% CI: 0.43–0.68) for PCNSL, and 0.56 (95% CI: 0.45–0.67) for studies including both. The corresponding forest plot of CR estimates is presented in **Figure 2**. No evidence of publication bias was detected using Egger’s test (p = 0.699) (**Figure 3**).

#### 3.4.4 Partial response

A total of 1,014 participants across 34 studies were included in the PR analysis. The pooled PR rate across all studies was 0.18 (95% CI: 0.14–0.22), with a prediction interval of 0.04 to 0.37. Moderate-to-substantial heterogeneity was observed among the included studies (I^2^ = 51.7%, τ^2^ = 0.0091, p = 0.0003). Subgroup analysis stratified by CNS lymphoma classification showed no significant difference in PR between PCNSL and SCNSL (p = 0.2910), with a pooled HR of 0.17 (95% CI: 0.11–0.23) for SCNSL, 0.11 (95% CI: 0.03–0.21) for PCNSL, and 0.21 (95% CI: 0.15–0.29) for studies including both. The PR forest plot is also shown in **Figure 2**. No evidence of publication bias was detected using Egger’s test (p = 0.64) (**Figure 3**).

#### 3.4.5 Meta-regression analysis

A mixed-effects multiple meta-regression analysis was conducted to assess the impact of CNS lymphoma classification and publication year on ORR. Publication year was not a significant moderator (p = 0.2254), indicating that efficacy outcomes have remained stable over the study period. Similarly, study design (p = 0.2308), PCNSL status (p = 0.1089) and SCNSL status (p = 0.0567) were not significantly associated with variations in ORR in **Table 3**.

**Table 3:** Mixed-effects meta-regression for objective response rate (ORR) including 38 studies (k = 38), fitted using a DerSimonian–Laird estimator.

Moderator	Estimate	SE	z-value	p-value	95% CI (Lower)	95% CI (Upper)
Intercept	−40.9906	34.7613	−1.1792	0.2383	−109.1215	27.1403
Observational design	−0.0771	0.0643	−1.1984	0.2308	−0.2032	0.0490
Publication year	0.0208	0.0172	1.2122	0.2254	−0.0128	0.0545
PCNSL group	−0.1460	0.0911	−1.6029	0.1089	−0.3245	0.0325
SCNSL group	−0.1027	0.0539	−1.9059	0.0567	−0.2083	0.0029

Residual heterogeneity was moderate (τ² = 0.0097; τ = 0.0987; I² = 50.1%; H² = 2.00), with moderators explaining 6.83% of heterogeneity (R²). Residual heterogeneity remained significant (QE(33) = 66.14, p = 0.0005). The omnibus test of moderators was not statistically significant (QM(4) = 8.35, p = 0.0795). PCNSL and SCNSL coefficients represent contrasts relative to the reference group. Reference: Design (Interventional); Group (Both).

### 3.5 Safety outcomes

#### 3.5.1 Cytokine release syndrome

The incidence of Cytokine Release Syndrome (CRS) was assessed among the included studies. The pooled incidence of any-grade CRS was 83.5% (95% CI: 78.7%–87.9%), with a prediction interval of 60.1% to 98.6%. The pooled incidence of Severe CRS (Grade ≥ 3) was 5.77% (95% CI: 2.98%–9.16%), with prediction interval was 0.0% to 23.4%. Substantial heterogeneity was observed for both any-grade CRS (I² = 64.3%) and Severe CRS (Grade ≥ 3) (I² = 61.8%). Detailed pooled estimates and heterogeneity statistics for CRS are summarized in **Table 4**. CRS rates stratified by CAR-T product are shown in **Supplementary Figure 5**. Product-specific random-effects estimates were 0.82 (95% CI: 0.77–0.86) for multi-product studies, 0.78 (95% CI: 0.44–0.99) for lisocabtagene maraleucel, 0.92 (95% CI: 0.73–1.00) for axicabtagene ciloleucel, and 0.80 (95% CI: 0.58–0.96) for tisagenlecleucel. Subgroup differences were not statistically significant under the random-effects model (p = 0.2335).

**Table 4:** Pooled incidence of cytokine release syndrome (CRS) and immune effector cell–associated neurotoxicity syndrome (ICANS) following CAR-T cell therapy.

Outcome	k	Observations (n)	Events (e)	Common-effect proportion (95% CI)	Random-effects proportion (95% CI)	Prediction interval	I² (%)
CRS (Any grade)	34	1,190	947	0.8238 (0.7991–0.8475)	0.8348 (0.7869–0.8786)	0.6010–0.9860	64.3
ICANS (Any grade)	31	1,048	425	0.3883 (0.3567–0.4202)	0.4486 (0.3596–0.5391)	0.0670–0.8660	84.2
CRS (Grade > 3)	33	1,180	98	0.0500 (0.0351–0.0665)	0.0577 (0.0298–0.0916)	0.0000–0.2341	61.8
ICANS (Grade > 3)	29	991	184	0.1530 (0.1284–0.1789)	0.1738 (0.1209–0.2327)	0.0036–0.4605	71.2

Proportions were synthesized using inverse-variance meta-analysis with Freeman–Tukey double arcsine transformation. Random-effects models were estimated using the DerSimonian–Laird method, with between-study heterogeneity quantified using τ², τ, I², and H statistics. Confidence intervals for τ² and τ were derived using the Jackson method. Prediction intervals were calculated using a t-distribution with degrees of freedom equal to k−1. Substantial heterogeneity was observed across outcomes, particularly for ICANS (any grade). All heterogeneity tests (Q statistic) were statistically significant (p < 0.0001).

#### 3.5.2 Immune effector cell-associated neurotoxicity syndrome

Neurotoxicity was assessed through the incidence of Immune Effector Cell-Associated Neurotoxicity Syndrome (ICANS). The overall pooled incidence of any-grade ICANS was 44.9% (95% CI: 36.0%–53.9%) with a prediction interval of 6.7% to 86.6%. The pooled incidence of Severe ICANS (Grade ≥ 3) was 17.4% (95% CI: 12.1%–23.3%) with a prediction interval of 0.36% to 46.1%. Substantial heterogeneity was observed for both any-grade ICANS (I^2^ = 84.2%) and Severe ICANS (Grade ≥ 3) (I^2^ = 71.2%). A comprehensive summary of ICANS incidence and heterogeneity metrics is provided in **Table 4**. ICANS rates stratified by CAR-T product are presented in **Supplementary Figure 6**. Product-specific random-effects estimates were 0.39 (95% CI: 0.29–0.49) for multi-product studies, 0.66 (95% CI: 0.54–0.76) for axicabtagene ciloleucel, 0.51 (95% CI: 0.33–0.70) for brexucabtagene autoleucel, 0.62 (95% CI: 0.17–0.99) for tisagenlecleucel, and 0.65 (95% CI: 0.24–0.97) for lisocabtagene maraleucel. Subgroup differences were statistically significant under the random-effects model (p < 0.0001).

### 3.6 Sensitivity analysis

**Figure 4:**
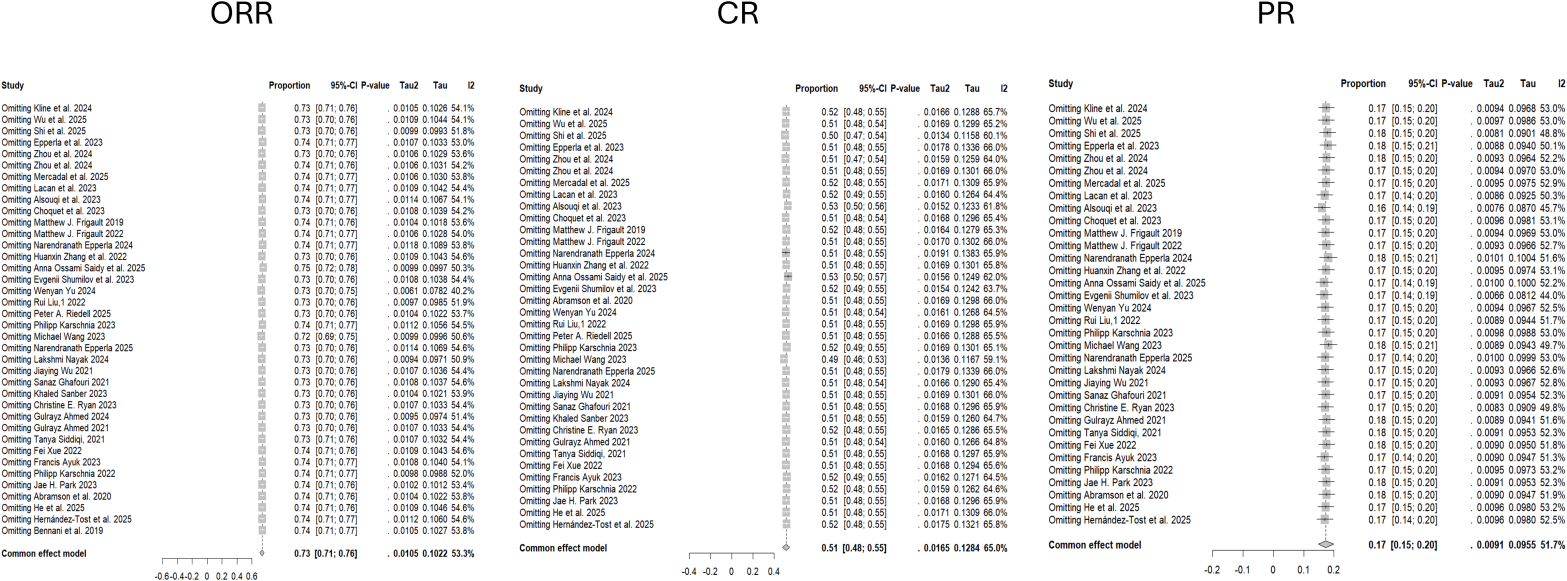
Leave-one-out sensitivity analyses for ORR, CR, and PR. ORR, Objective Response Rate; CR, Complete Response; PR, Partial Response.

The leave-one-out sensitivity analysis demonstrates solid and robust results, as sequential omission of each study produces only negligible changes in the pooled proportions, confidence intervals, and heterogeneity estimates across all three models, indicating that no single study unduly influences the overall findings; the pooled estimates remain highly stable (approximately 0.73, 0.51, and 0.17, respectively) (**Figure 4**).

## 4 Discussion

This study represents a comprehensive assessment of CAR T-cell therapy, a novel T-cell based immunotherapy targeting B-cell malignancies, in patients with CNS lymphoma. Our study included patients across 39 studies. Overall, CAR T-cell therapy demonstrated robust therapeutic efficacy, with an ORR of 75%, and a CR achieved in 52% of patients receiving treatment. Additionally, subgroup analysis revealed no significant difference in outcomes between SCNSL and PCNSL, suggesting that CAR T-cell therapy offers robust benefit regardless of disease origin. However, despite treatment effectiveness, the pooled safety assessment revealed that 83.5% and 44.9% of patients developed CRS and ICANS respectively. Additionally, only 5.8% and 17.4% of these patients were classified as severe CRS or ICANS, indicating a high prevalence of immune-mediated toxicities in patients receiving CAR T-cell therapy, without a high frequency of high-grade events. Regarding the mixed-effects multiple meta-regression analysis, the absence of a temporal effect of publication year on ORR indicates that overall efficacy has remained stable over the period captured by this analysis, despite advances in clinical experience and supportive care.

Overall, this study reports an efficacy profile consistent with previous literature. A previous review by Cook et al. included 128 patients across 15 studies, and reported remarkably similar results (ORR: 74%, CR: 56% for PCNSL; ORR: 63%, CR: 47% for SCNSL). (Cook et al., 2023) Notably, our subgroup analyses found no statistically significant differences between PCNSL and SCNSL in terms of ORR or CR. Our results, including a much larger and more heterogeneous dataset, strengthen the evidence that CAR T-cell efficacy is preserved across CNS lymphoma subtype, which due to the universal expression of the CD19 antigen across these B-cell malignancies, and the ability of CAR T-cells to effectively penetrate the blood-brain barrier regardless of disease classification (54, 55). Regarding safety, Cook et al. reported similar toxicity profiles for CRS (Any Grade: 70-72%, Grade ≥ 3: 11-13%) and ICANS (Any Grade: 48-53%, Grade ≥ 3: 18-26%) across PCNSL and SCNSL patients (Cook et al., 2023), aligning with this studies pooled data.

Additionally, our results are also consistent with the landmark CAR T-cell therapy registration trials for systemic large B-cell lymphoma (LBCL). Specifically, our pooled severe (Grade ≥ 3) ICANS rate of 16.8% falls within the range reported in the ZUMA-1 (28%) (56), JULIET (12%) (57), and TRANSCEND (10%) trials (7). Additionally, the incidence of severe CRS in our cohort (5.7%) was notably lower than that observed in the ZUMA-1 (13%) (56) and JULIET (22%) cohorts (57). however higher than the TRANSCEND cohort (7), suggesting that the immune-mediated toxicities observed are expected and clinically manageable. The relatively lower rate of severe CRS compared with the ZUMA-1 and JULIET trials may reflect differences in patient selection, disease burden, or toxicity management strategies. Consistent with previous studies, our data suggests that CNS disease localization does not inherently predispose patients to an increased risk of severe ICANS, suggesting that the presence of CNS disease should not be an automatic barrier to CAR T-cell use when appropriate monitoring and management are available (36, 58). To contextualize these findings, it is important to consider survival outcomes observed with standard salvage strategies in relapsed CNS lymphoma. Conventional standard-of-care (SOC) approaches (eg. high-dose methotrexate, cytarabine, targeted agents, or whole-brain radiotherapy regimens) have generally yielded short-lived response; Retrospective data highlight the dismal outcomes of relapsed CNS lymphoma, with the French oculo-cerebral lymphoma network reporting on >1,000 patients that approximately 25% were primary refractory to methotrexate (MTX)-based therapy and another 25% relapsed, resulting in a median overall survival of just 6.8 months from relapse; although early-phase studies showed that ibrutinib achieves therapeutic CNS penetration and, when combined with R-MTX induction, induced responses in 12 of 15 patients with a median PFS of 4.8 months, and immunomodulatory agents (± rituximab) produced response rates of 50–60%, durability remained limited with median PFS of only 4–5 months, underscoring the continued unmet need in this population (59). In the cohort by Choquet et al. (48), CAR T-cell therapy was associated with a significantly prolonged median OS and PFS (mOS: 21.2, mPFS: 8.4) compared with a control group treated with SOC therapies (mOS: 3.0, mPFS: 4.7). Our pooled median-of-median OS estimates were concordant with the survival outcomes observed in the CAR T-cell cohort of Choquet et al., and appear longer than those historically achieved with SOC alone.

This study is constrained by several limitations. We observed significant statistical heterogeneity across the included studies, particularly regarding complete response (I^2^ = 65.0%), ICANS (I^2^ = 84.2%), and CRS (I^2^ = 64.3%) rates. Our meta-regression analysis indicated that this variability was not driven by the year of publication (p = 0.2254), suggesting that efficacy has remained stable over time. Instead, this heterogeneity likely stems from differences in clinical management and CAR T-cell product characteristics. Notably, CAR T-cell therapies utilizing a CD28 costimulatory domain (e.g., Axicabtagene Ciloleucel) have been associated with higher rates of ICANS compared to those using 4-1BB domains (14), and variations in bridging therapies ranging from systemic chemotherapy to whole-brain radiation further contribute to the diverse clinical landscape. Additionally, this study also included both retrospective and prospective data, introducing unavoidable heterogeneity. However, despite the limitations inherent to the nature of this meta-analysis, it is strengthened by its large cumulative sample size and the absence of detectable publication bias, providing the most robust evidence to date for the clinical utility of CAR T-cell therapy in this population. Future investigations should include standardized reporting and consequent sub-analysis of CNS disease compartments (e.g., parenchymal, leptomeningeal, ocular, or dural involvement), bridging therapy, and CART-cell therapy product (e.g., axi-cel, tisa-cel, or liso-cel) to better outline the impact of these variables on treatment outcomes. Additionally, new emerging strategies should be explored, such as localized or intrathecal CAR T delivery for CNS lymphoma, which aims to optimize CNS exposure and potentially decrease systemic toxicity (60, 61).

## 5 Conclusion

Our analysis reveals CAR T-cell therapy as a promising treatment for CNS lymphoma, with comparable efficacy in PCNSL and SCNSL. Despite data variability, the analysis shows a high incidence of CRS, and ICANS associated with CAR-T treatment, but both are manageable. Future papers should be directed towards improving the treatment’s safety, by recommending new guidelines for the subsets at risk of developing severe neurotoxicity. Continuous research on CAR-T therapy is required to improve treatment of CNS lymphomas.

## Data Availability

Relevant data (e.g. extraction sheets) are available upon request from the authors.

## Supplementary material

Supplementary Figure 1 Forest plot of pooled hazard ratios (HRs) for progression-free survival (PFS) comparing primary CNS lymphoma (PCNSL) versus secondary CNS lymphoma (SCNSL). Individual study HRs with 95% confidence intervals are shown, along with common-effect and random-effects pooled estimates. Values <1 favor improved prognosis for PCNSL relative to SCNSL. Heterogeneity statistics (I², τ², and p-value) are reported.

Supplementary Figure 2 Forest plot of pooled overall survival (OS) rates stratified by follow-up time point (6, 12, and 24 months). Individual study proportions with 95% confidence intervals are displayed, with marker size proportional to study weight. Common-effect and random-effects pooled estimates are shown for each subgroup and overall. Heterogeneity statistics (I², τ², and p-values), subgroup comparison tests, and prediction intervals for the random-effects model are reported.

Supplementary Figure 3 Forest plot of pooled progression-free survival (PFS) rates stratified by follow-up time point (3, 6, 9, and 12 months). Individual study proportions with 95% confidence intervals are shown, along with common-effect and random-effects pooled estimates. Overall pooled PFS rates and subgroup heterogeneity statistics (I² and p-values) are presented. Prediction intervals are displayed for random-effects models where applicable.

Supplementary Figure 4 Forest plot of pooled objective response rates (ORR) stratified by CAR-T product (multiple products, tisa-cel, liso-cel, Yescarta, axi-cel, brexu-cel, and other/NR). Multiple: Studies including ≥2 CAR-T products (e.g., Axi-cel + Tisa-cel + Liso-cel ± Brexu-cel); Tisa-cel: Axicabtagene ciloleucel only; Liso-cel: Lisocabtagene maraleucel only; Axi-cel: Axicabtagene ciloleucel only; Brexu-cel: Brexucabtagene autoleucel only; Other/NR: CD19-directed (generic/unspecified); 4-1BB–based targeting CD19/CD20/CD22, 1928ζ-CD19 CAR.

Supplementary Figure 5 Forest plot of pooled cytokine release syndrome (CRS) stratified by CAR-T product (multiple products, tisa-cel, liso-cel, Yescarta, axi-cel, brexu-cel, and other/NR). Multiple: Studies including ≥2 CAR-T products (e.g., Axi-cel + Tisa-cel + Liso-cel ± Brexu-cel); Tisa-cel: Axicabtagene ciloleucel only; Liso-cel: Lisocabtagene maraleucel only; Axi-cel: Axicabtagene ciloleucel only; Brexu-cel: Brexucabtagene autoleucel only; Other/NR: CD19-directed (generic/unspecified); 4-1BB–based targeting CD19/CD20/CD22, 1928ζ-CD19 CAR.

Supplementary Figure 6 Forest plot of pooled Immune Effector Cell–Associated Neurotoxicity Syndrome (ICANS) stratified by CAR-T product (multiple products, tisa-cel, liso-cel, Yescarta, axi-cel, brexu-cel, and other/NR). Multiple: Studies including ≥2 CAR-T products (e.g., Axi-cel + Tisa-cel + Liso-cel ± Brexu-cel); Tisa-cel: Axicabtagene ciloleucel only; Liso-cel: Lisocabtagene maraleucel only; Axi-cel: Axicabtagene ciloleucel only; Brexu-cel: Brexucabtagene autoleucel only; Other/NR: CD19-directed (generic/unspecified); 4-1BB–based targeting CD19/CD20/CD22, 1928ζ-CD19 CAR.
