# Supplementary material for: Safety and efficacy of CAR T-cell therapy in central nervous system lymphoma: a systematic review and meta-analysis
Source: Front Oncol. 2026 Mar 23;16:1790444. doi: 10.3389/fonc.2026.1790444 (PMC13050687; doi:10.3389/fonc.2026.1790444)
Supplement: Supplementary file 1 [file Table1.docx]

Contents

[**Supplementary Table 1 – Search Strategy (Table S1)** 2](#_Toc223216779)

[**Methodology** 3](#_Toc223216780)

[**Supplementary Table 2.2 – Baseline characteristics 2 (Table S2.2)** 5](#_Toc223216781)

[**Supplementary Table 3 – Quality assessment for single-arm retrospective cohort studies (Table S3)** 6](#_Toc223216782)

[**Supplementary Table 4 – Quality assessment for single-arm interventional studies (Table S4)** 7](#_Toc223216783)

[**Supplementary Table 5 – Median survival outcomes in CAR T-Cell therapy for CNS lymphoma (Table S5)** 8](#_Toc223216784)

[**Supplementary Table 6 – Distribution of CAR T-Cell Products and Target Constructs Stratified by Population in Included Studies (Table S6)** 9](#_Toc223216785)

[**Supplementary Table 7 – Cross-Study Frequency and Geographic Distribution of CAR T-Cell Products in CNS Lymphoma Studies (Table S7)** 9](#_Toc223216786)

[**References** 10](#_Toc223216787)

| 1 | "Chimeric* antigen receptor T cell*" OR "CAR-T cell*" OR CAR-T OR "CAR Therapy" OR Axicabtagene ciloleucel OR Axi-cel OR KTE-C19 OR KTEC19 OR CTL-019 OR CTL019 OR Yescarta OR Lisocabtagene maraleucel OR Liso-cel OR JCAR-017 OR JCAR017 OR Breyanzi OR Brexucabtagene autoleucel OR Brexu-cel OR KTE-X19 OR KTEX19 OR Tecartus OR Tisagenlecleucel OR Tisa-cel OR Kymriah OR CART-19 OR CART19 |
| --- | --- |
| 2 | central nervous system OR CNS AND lymphoma* |
| 3 | "Primary CNS lymphoma*" OR "Secondary CNS lymphoma*" OR "Primary central nervous system lymphoma*" OR "Secondary central nervous system lymphoma*" OR PCNSL OR SCNSL OR "CNS lymphoma*" |
| 4 | (Central Nervous System Neoplasms OR Central Nervous System OR Nervous System Diseases OR Central Nervous System Diseases) AND (Lymphoma OR "Lymphoma, Non-Hodgkin" OR exp Lymphoma, B-Cell OR Burkitt Lymphoma OR "Lymphoma, Large B-Cell, Diffuse" OR exp Lymphoma, TCell) |
| 5 | #2 OR #3 OR #4 |
| 6 | #1 AND #5 |

# **Supplementary Table 1 – Search Strategy (Table S1)**

Search strategy for Medline is presented, search strategies for other databases were modified according to each database.

#

# **Methodology**

**Data Extraction**

Data extraction was performed independently by two reviewers using a standardized, pilot-tested data extraction form developed in Microsoft Excel. The following information was extracted from each included study: (1) study characteristics: first author name, year of publication, country or countries of origin, study design (prospective vs. retrospective, phase of clinical trial if applicable), sample size, and duration of follow-up; (2) patient demographics and disease characteristics: median or mean age, sex distribution, CNS lymphoma classification (PCNSL, SCNSL, or both), histologic subtype (e.g., diffuse large B-cell lymphoma), sites of CNS involvement (e.g., parenchymal, leptomeningeal, intraocular), and number of prior lines of therapy; (3) CAR T-cell product characteristics: specific CAR T-cell product (axicabtagene ciloleucel, tisagenlecleucel, lisocabtagene maraleucel, or other), and CAR T-cell dose; (4) treatment details: bridging therapy administered (yes/no and type if reported), and conditioning chemotherapy regimen; (5) efficacy outcomes: number of patients achieving overall response (complete response plus partial response), complete response, and partial response, assessed post-infusion or best response during follow-up; and (6) safety outcomes: number of patients experiencing any-grade cytokine release syndrome (CRS) and grade ≥3 CRS, and any-grade immune effector cell-associated neurotoxicity syndrome (ICANS) and grade ≥3 ICANS, graded according to the American Society for Transplantation and Cellular Therapy (ASTCT) consensus grading criteria or equivalent.

For studies that reported outcomes at multiple time points, data from the time point with the longest follow-up were extracted. When outcomes were reported separately for PCNSL and SCNSL subgroups within a single study, data for each subgroup was extracted separately. Discrepancies in data extraction were identified through comparison and resolved through discussion with a third reviewer if necessary.

**Quality Assessment**

The methodological quality and risk of bias of included studies were assessed independently by two reviewers using validated quality assessment tools appropriate for the specific study designs.

For single-arm retrospective cohort studies n=28, a modified version of the Newcastle-Ottawa Scale (NOS) was utilized. (1) Given the single-arm nature of these studies, the standard "Comparability" domain was excluded. This modified scale evaluated studies across two domains with a maximum possible total score of 6 stars, the quality of the included cohort studies was assessed using the Newcastle-Ottawa Scale (NOS). Under the Selection domain (maximum of three stars), one star was assigned for the representativeness of the exposed cohort, one for the ascertainment of exposure, and one for the demonstration that the outcome of interest was not present at the start of the study. Under the Outcome domain (maximum of three stars), one star was awarded for the assessment of outcome, one for adequate follow-up duration to allow outcomes to occur, and one for the adequacy of cohort follow-up.

One star was awarded for each satisfied criterion. Follow-up duration was deemed adequate if it allowed for the assessment of acute toxicities and initial responses (typically ≥1 month post-infusion), as most events occur early after CAR T-cell therapy. Based on this modified scale, studies with a total score of 5-6 stars were classified as good quality, while studies with a score of ≤4 stars were classified as moderate quality. The detailed NOS assessments for all cohort studies are provided in the supplementary **[Table-S3].**

For single-arm interventional studies (n=11), including phase I and phase II clinical trials and prospective case series, the Methodological Index for Non-Randomized Studies (MINORS) was applied. (2) MINORS consists of 8 items. Each item was scored as 0 (not reported), 1 (reported but inadequate), or 2 (reported and adequate), with a maximum possible score of 16 for non-comparative studies. The following domains were assessed: (1) a clearly stated aim, (2) inclusion of consecutive patients, (3) prospective collection of data, (4) endpoints appropriate to the aim of the study, (5) unbiased assessment of the study endpoint, (6) follow-up period appropriate to the aim of the study, (7) loss to follow-up less than 5%, and (8) prospective calculation of the study size. Studies with scores ≥12 (of 16) were considered good quality, and studies scoring 10-11 were classified as moderate quality. The detailed MINORS assessments for all interventional studies are provided in Supplementary **[Table-S4]**.

Quality assessment focused on the following key methodological aspects: clarity of study aims and objectives, clarity of inclusion and exclusion criteria, prospective or retrospective data collection, appropriate outcome assessment methods, adequate follow-up duration, reporting of loss to follow-up, and appropriate statistical analysis. Disagreements in quality assessment were resolved by consensus or by consulting a third senior reviewer. Studies were not excluded based on quality assessment scores; however, quality scores were considered in sensitivity analyses and in the interpretation of results.

| **Author** | **Year of Publication** | **Type of Study** | **Location of Study** | **Median Follow Up (Months)** | **Reported Safety Outcomes** | **Reported Efficacy Outcomes** |
| --- | --- | --- | --- | --- | --- | --- |
|  |  |  |  |  |  |  |
| **Kline et al.** | 2024 | Prospective case series | USA | 2.9 | ICANS any grade; ICANS ≥3; HLH any grade | ORR, CR, PR, PFS, OS |
| **Wu et al.** | 2025 | Retrospective cohort | China | 37.5 | CRS any grade; CRS ≥3; ICANS any grade; ICANS ≥3; cytopenias ≥3; infections ≥3 | ORR, CR, PR, PFS, HR-PFS, OS, HR-OS, DOR, OS rate, PFS rate |
| **Shi et al.** | 2025 | Retrospective cohort | China | 12 | CRS any grade; ICANS any grade; ICANS ≥3 | ORR, CR, PR, PFS, HR-PFS, OS, HR-OS, OS rate, PFS rate |
| **3Epperla et al.** | 2023 | Retrospective cohort | USA | 14.1 | CRS any grade; CRS ≥3; ICANS any grade; ICANS ≥3 | ORR, CR, PR, PFS, HR-PFS, OS, DOR, OS rate, PFS rate |
| **Zhou et al.** | 2024 | Retrospective cohort | China | 16.73 | CRS any grade; CRS ≥3; ICANS any grade; cytopenias ≥3; infections ≥3 | ORR, CR, PR, PFS, OS, OS rate, PFS rate |
| **Mercadal et al.** | 2025 | Retrospective cohort | USA | 26 | CRS any grade; ICANS any grade; ICANS ≥3 | ORR, CR, PR, PFS, OS, OS rate, PFS rate |
| **Lacan et al.** | 2023 | Retrospective cohort | France | 12 | CRS any grade; CRS ≥3; ICANS any grade; ICANS ≥3 | ORR, CR, PR, PFS, OS, OS rate, PFS rate |
| **Alsouqi et al.** | 2023 | Retrospective cohort | USA | 10.7 | CRS any grade; CRS ≥3; ICANS any grade; ICANS ≥3 | ORR, CR, PR, PFS, HR-PFS, OS, HR-OS, OS rate, PFS rate |
| **Choquet et al.** | 2023 | Retrospective cohort | France | 20.8 | CRS any grade; CRS ≥3; ICANS any grade; ICANS ≥3; cytopenias ≥3; neurotoxicity any grade | ORR, CR, PR, PFS, OS, OS rate, PFS rate |
| **Frigault et al.** | 2019 | Retrospective cohort | USA | NR | CRS any grade | ORR, CR, PR |
| **Alcantara et al.** | 2022 | Retrospective cohort | France | 8.5 | CRS any grade; CRS ≥3; ICANS any grade; ICANS ≥3; cytopenias ≥3 (prolonged) | ORR, CR, PR, PFS, OS |
| **Frigault et al.** | 2022 | Prospective phase 1/2 | USA | 12.2 | CRS any grade; ICANS any grade; ICANS ≥3 | ORR, CR, PR, PFS, OS |
| **Epperla et al.** | 2024 | Retrospective Cohort | N/A | 2 | CRS any grade; CRS ≥3; ICANS any grade; ICANS ≥3 | ORR, CR, PR, PFS, HR-PFS, OS, HR-OS, OS rate, PFS rate |
| **Lin et al.** | 2024 | Retrospective cohort | China | NR | CRS any grade; CRS ≥3; ICANS any grade; ICANS ≥3; cytopenias ≥3; infections ≥3 | NR |
| **Zhang et al.** | 2022 | Retrospective cohort | China | 12 | CRS any grade; ICANS any grade; ICANS ≥3; cytopenias ≥3; organ toxicity (GI, liver, renal, cardiac) any grade | ORR, CR, PR, PFS, OS, OS rate, PFS rate |
| **Saidy et al.** | 2025 | Retrospective cohort | EU | 24.8 | CRS any grade; CRS ≥3; ICANS any grade; ICANS ≥3; cytopenias any grade; infections ≥3; deaths from infection | ORR, CR, PR, PFS, OS, HR-PFS, HR-OS |
| **Shumilov et al.** | 2023 | Retrospective cohort | Switzerland | 4.9 | CRS any grade; CRS ≥3; ICANS any grade; ICANS ≥3; HLH any grade; cytopenias any grade; infections any grade; deaths from infection | ORR, CR, PR, PFS, OS |
| **Yu et al.** | 2024 | Retrospective cohort | China | 10.5 | CRS any grade; CRS ≥3; ICANS any grade; ICANS ≥3; cytopenias any grade; infections any grade; hepatic dysfunction any grade | ORR, CR, PR, OS rate |
| **Liu et al.** | 2022 | Prospective Clinical Trial, Single-Arm, Single-Center (Nonrandomized) | China | 10.4 | CRS any grade; ICANS any grade; cytopenias any grade | ORR, CR, PR |
| **Riedell et al.** | 2025 | Retrospective cohort | USA | 15.5 | ICANS any grade; ICANS ≥3 | ORR, CR, PR |
| **Karschnia et al.** | 2023 | Retrospective cohort | USA | 12.0 | CRS any grade; CRS ≥3; ICANS any grade; ICANS ≥3 | ORR, CR, PR, PFS, OS |
| **Wang et al.** | 2023 | Phase I, open-label, multicenter, seamless design | USA | 16.1 | CRS any grade; CRS ≥3; ICANS any grade; ICANS ≥3; cytopenias ≥3; infections ≥3; hypogammaglobinemia any grade | ORR, CR, PR, PFS, OS, OS rate, PFS rate |
| **Epperla et al.** | 2025 | Multicenter retrospective real-world cohort study | USA | 48.2 | CRS any grade; CRS ≥3; ICANS any grade; ICANS ≥3 | ORR, CR, PR, PFS, OS, OS rate, PFS rate |
| **Nayak et al.** | 2024 | Pilot study, single-arm, phase I/II style (safety & efficacy endpoints | USA | 24.2 | CRS any grade; ICANS any grade; ICANS ≥3 | ORR, CR, PR, PFS, OS, OS rate, PFS rate |
| **Wu et al.** | 2021 | Single-center, open-label, single-arm clinical trial | China | 14.2 | CRS any grade; ICANS any grade; ICANS ≥3; infections any grade | ORR, CR, PR, OS rate, PFS rate |
| **Ghafouri et al.** | 2021 | Retrospective, single-institution case series | USA | 11.5 | CRS any grade; ICANS any grade; ICANS ≥3; infections any grade | ORR, CR, PR, PFS, OS |
| **Sanber et al.** | 2023 | Retrospective cohort | USA | 13.8 | CRS any grade; ICANS any grade | ORR, CR, PR |
| **Ryan et al.** | 2023 | Retrospective cohort | USA | 15.4 | CRS any grade; CRS ≥3; ICANS any grade; ICANS ≥3 | ORR, CR, PR, OS rate, PFS rate |
| **Ahmed et al.** | 2024 | Retrospective cohort | USA | 16.7 | NR | ORR, CR, PR, OS rate, PFS rate |
| **Ahmed et al.** | 2021 | Retrospective cohort | USA | 5.1 | CRS any grade; CRS ≥3; ICANS any grade; ICANS ≥3 | ORR, CR, PR, PFS, OS |
| **Siddiqi et al.** | 2021 | Phase 1 clinical trial | USA | NR | CRS any grade; CRS ≥3 | ORR, CR, PR, OS |

**Table 2.1** provides a concise overview of study design, geographic setting, follow-up duration, and the spectrum of reported safety and efficacy endpoints for included CAR T-cell studies in CNS lymphoma. It highlights the predominance of retrospective cohorts from the USA, China, and Europe, with variable follow-up but consistently detailed reporting of CRS, ICANS, cytopenias, infections, and standard lymphoma efficacy outcomes (ORR, CR, PR, PFS, OS).

**Supplementary Table 2.1 – Baseline Characteristics (Table S2.1)**

**NR -**  Not Reported; S **-**  Survived; **D -** Deceased

| **Author** | **Year of Publication** | **Population** | **Analyzed/Total number of patients** | **Median age** | **M:F** | **Tumor Histology** | **CAR-T Cell Product** | **Prior ASCT n(%)** |
| --- | --- | --- | --- | --- | --- | --- | --- | --- |
| **Kline et al.** | 2024 | SCNSL | 4/4 | 38.3 | 4:0 | Burkitt lymphoma (2/4)  Non-GCB DLBCL (1/4)  HGBCL (1/4) | Axi-cel, Tisa-cel, Liso-cel | 1 (25) |
| **Wu et al.** | 2025 | Both | 38/38 | 47 | 23:15 | DLBCL (36/38, 94.7%),  Burkitt's lymphoma (1/38, 2.6%),  Intravascular large B-cell Lymphoma (1/38, 2.6%) | Axi-cel, Relma-cel | 2 (5.3) |
| **Shi et al.** | 2025 | Both | 27/27 | 58 | 15:12 | PCNSL 19/27 (70.4%)  SCNSL 8/27 (29.6%)  DLBCL 5/27 (18.5%)  PMBL 1/27 (3.7%)  BL 1/27 (3.7%)  Richter's 1/27 (3.7%) | CD19 Directed  CD20 Directed  CD19/CD22 Directed | 6 (22.2) |
| **Epperla et al.** | 2023 | SCNSL | 61/61 | 56 | 34:27 | De novo DLBCL (n=48/61), Transformed Lymphoma (n=7/61), Others (n=4/61) | Axi-cel, Tisa-cel, Liso-cel, Brexu-cel | 14 (23) |
| **Zhou et al.** | 2024 | Both | ASCT +CART 29/29  CART 10/10  CIT 17/17 | 42   38.5  62 | 14:15   3:7   10:7 | DLBCL (29/29)   DLBCL (10/10)   DLBCL (17/17) | CD19/CD22 Directed | 25 (100), 0 (0) |
| **Mercadal et al.** | 2025 | PCNSL | 24/24 | 57 | 16:8 | DLBCL (23/23) | Axi-cel, Tisa-cel | 12 (50) |
| **Lacan et al.** | 2023 | Both | 21/21 | 67 | 11:10 | NR | Axi-cel, Tisa-cel | 16 (76.2) |
| **Alsouqi et al.** | 2023 | SCNSL | 80/86 | 62 | 53:33 | DLBCL (N = 67 (78%)), tFL (N = 12 (14%)) HGBCL (N = 6 (7.0%) Burkitt lymphoma (N = 1 (1.2%)). | Axi-cel, Tisa-cel, Liso-cel | 22 (19) |
| **Choquet et al.** | 2023 | PCNSL | 27/247 | 68 | 14:13 | DLBCL (27/27) | Axi-cel, Tisa-cel | 14 (52) |
| **Matthew J. Frigault** | 2019 | SCNSL | 8/8 | 50 | 4:4 | DLBCL (5/8)  PMBCL(1/8)  HGBCL(2/8) | Tisa-cel | 1 (12.5) |
| **Marion Alcantara** | 2022 | PCNSL | 9/9 | 67 | 3:6 | NR | Axi-cel, Tisa-cel | 7 (77.8) |
| **Matthew J. Frigault** | 2022 | PCNSL | 12/12 | 63 | 7:5 | LBCL (12/12) | Tisa-cel | 3 (25) |
| **Narendranath Epperla** | 2024 | SCNSL | 136/144 | 61 | 52:92 | NR | Axi-cel, Tisa-cel, Liso-cel | 44 (31) |
| **Haolong Lin et al.** | 2024 | SCNSL | ASCT+CART: 23/26  CART: 3/26 | NR | NR | BCL (26/26) | CD19 Directed  CD22 Directed  CD30 Directed  CD19/22 Directed  CD19/20 Directed  CD20/22 Directed  CD19/20/22 Directed | 23 (88.5) |
| **Huanxin Zhang et al.** | 2022 | SCNSL | 15/15 | 51 | 11:4 | DLBCL (13/15) / PMBCL (1/15)/ burkitt (1/15) | CD19 Directed  CD19/20 Directed  CD19/22 Directed | 2 (10.5) |
| **Anna Ossami Saidy et al.** | 2025 | Both | 100/106 | 62 | 62:38 | DLBCL 86 (86)   High‐grade lymphoma (Myc + Bcl‐2 ± Bcl‐6) 6 (6), others 8 (8) | Axi-cel, Tisa-cel, Brexu-cel | 40 (40) |
| **Evgenii Shumilov et al.** | 2023 | Both | 15/15 | 61 | 8:7 | DLBCL 15 (100) de novo 13 | Axi-cel, Tisa-cel | 9 (60) |
| **Wenyan Yu** | 2024 | Both | 22/22 | 56 | 14:8 | DLBCL GCB 13 (59.1)  DLBCL non-GCB 9 (40.9)  DHL/THL 4 (18.2) | Relma-cel | 1 (4.5) |
| **Rui Liu,1** | 2022 | Both | 7/7 | 48 | 3:4 | DLBCL 7 (100%) | CD19 Directed  CD20 Directed | 2 (28.6) |
| **Peter A. Riedell** | 2025 | SCNSL | 10/101 | NR | NR | NR | Liso-cel | NR |
| **Philipp Karschnia** | 2023 | Both | 45/45 | 32 | 26:19 | DLBCL 37 (82.2%) Burkitt’s lymphoma           1 (2.2%) Transformed lymphoma 9(20%) | Axi-cel, Tisa-cel, Liso-cel | 9 (20.0) |
| **Michael Wang** | 2023 | SCNSL | 7/88 | 68.5 | 67:21 | Mantle Cell Lymphoma | Liso-cel | 29 (33) |
| **Narendranath Epperla** | 2025 | SCNSL | 65/65 | 63 | 43:22 | DLBCL (all included); subset with double-/triple-hit lymphoma 12/50 (24%) | Axi-cel | 12(18) |
| **Lakshmi Nayak** | 2024 | Both | 17/18 | 62 | 10:8 | Diffuse large B-cell lymphoma (DLBCL) involving CNS | Tisa-cel | NR |
| **Jiaying Wu** | 2021 | Both | 4/13 | 42 | 6:7 | All DLBCL (Diffuse Large B-cell Lymphoma), incl. 4 Primary CNSL, 9 Secondary CNSL | BrexU-cel | 0 |
| **Sanaz Ghafouri** | 2021 | SCNSL | 5/5 | 63 | 3:2 | All DLBCL | Axi-cel | 1 (20) |
| **Khaled Sanber** | 2023 | Both | 3/3 | 45 | 2:1 | DLBCL 1/3  B-ALL (Ph-) 1/3  NS 1/3 | Axi-cel, Brexu-cel | 1 (33.3) |
| **Christine E. Ryan** | 2023 | SCNSL | 7/7 | 60.1 | 4:3 | MCL (7/7):  Blastoid 3/7  Classic 1/7  Ploeomorphic 1/7  NS 2/7 | Tisa-cel, Brexu-cel | 2 (28.6) |
| **Gulrayz Ahmed** | 2024 | SCNSL | 8/8 | 72 | 1:7 | Mantle cell lymphoma (MCL) for all cases (no other lymphoma types in this cohort) | Brexu-cel | NR |
| **Gulrayz Ahmed** | 2021 | SCNSL | 7/7 | 50 | 4:3 | DLBCL | Axi-cel, Tisa-cel | NR |
| **Tanya Siddiqi,** | 2021 | PCNSL | 5/5 | 49 | 0:5 | DLBCL | CD19 Directed | NR |
| **Fei Xue** | 2022 | Both | 17/17 | 42 | 9:8 | DLBCL (15/17), MCL (1/17), BL (1/17) | 4-1BB Based Targeting CD19/CD20/CD22 | 3(17.6) |
| **Francis Ayuk** | 2023 | SCNSL | 28/28 | 58 | 16:12 | N/A | Axi-cel, Tisa-cel | NR |
| **Philipp Karschnia** | 2022 | SCNSL | 10/10 | 55 | 6:4 | DLBCL (6), trFL (3), PTLD (1) | CD19  Directed | NR |
| **Jae H. Park** | 2023 | SCNSL | 5/5 | 64 | 2:3 | DLBCL 2/5  HGBCL 2/5  MCL 1/5 | Axi-cel, Tisa-cel, Brexu-cel | 3(60) |
| **Jeremy S Abramson** | 2020 | SCNSL | 6/7 | NR | NR | NR | Liso-cel | NR |
| **He et al.** | 2025 | SCNSL | 21/21 | NR | 9:12 | B-ALL (11), B-NHL (10): DLBCL (7), Burkitt (1), HGBL (1), B-LBL (1). | 1928zT2 cells (3^rd^ Generation CAR-T) | 8(38) |
| **Hernández-Tost et al.** | 2025 | Both | 48/48 | 62 | 27:21 | DLBCL (46), Mantle Cell Lymphoma (2). | Axi-cel, Tisa-cel, Liso-cel | 26(54) |
| **Bennani et al.** | 2019 | SCNSL | 15/17 | 58 | 11:6 | B-cell NHL | Axi-cel | 7(41) |

**Table 1.2:** Demographic and Clinical Characteristics of Patients Treated with CAR T-Cell Therapy for CNS Lymphoma. The summarizes baseline characteristics from 39 studies (2019-2025) involving 1,457 patients treated with CAR T-cell therapy for primary or secondary CNS lymphoma. Data extracted include patient demographics (age, sex), CNS lymphoma type, tumor histology, CAR T-cell product administered, and prior autologous stem cell transplantation (ASCT) exposure. Diffuse large B-cell lymphoma (DLBCL) was the predominant histology. Median age ranged from 32-72 years, and prior ASCT exposure varied from 0-100%, reflecting heterogeneous patient populations across studies.

**Abbreviations:** ASCT, autologous stem cell transplantation; CAR T, chimeric antigen receptor T-cell; DLBCL, diffuse large B-cell lymphoma; NR, not reported; PCNSL, primary CNS lymphoma; SCNSL, secondary CNS lymphoma.

# **Supplementary Table 2.2 – Baseline characteristics 2 (Table S2.2)**

**NR -**  Not Reported.

| Author Name (Year) | Modified Newcastle-Ottowa scale for cohort | | | | | | |
| --- | --- | --- | --- | --- | --- | --- | --- |
|  | Selection | | | Outcome | | | Score (Out of 6) |
|  | Representativeness of the exposed cohort (*) | Ascertainment of exposure (*) | Demonstration that outcome of interest was not present at start of study (*) | Assessment of outcome (*) | Was follow-up long enough for outcomes to occur (*) | Adequacy of follow up of cohorts (*) |  |
| Wu et al. (2025) | * | * | * | * | * | * | 6 |
| Shi et al. (2025) | * | * | * | * | * | * | 6 |
| Epperla et al. (2023) | * | * | * | * | * | * | 6 |
| Zhou et al. (2024) | * | * | * | * | * | X | 5 |
| Mercadal et al. (2025) | * | * | * | * | * | X | 5 |
| Lacan et al. (2023) | * | * | * | * | * | X | 5 |
| Alsouqi et al. (2023) | * | * | * | * | * | X | 5 |
| Choquet et al. (2023) | X | * | * | * | * | * | 5 |
| Frigault et al. (2019) | * | * | * | * | X | * | 5 |
| Alcantara et al. (2022) | * | * | * | * | * | * | 6 |
| Epperla et al. (2024) | * | * | * | * | * | * | 6 |
| Lin et al. (2024) | * | * | * | * | * | * | 6 |
| Zhang et al. (2022) | * | * | * | * | * | * | 6 |
| Saidy et al. (2025) | * | * | * | * | * | * | 6 |
| Shumilov et al. (2023) | * | * |  | * | X | X | 4 |
| Yu et al. (2024) | * | * | * | * | * | * | 6 |
| Riedell et al. (2025) | * | * | * | * | * | * | 6 |
| Karschnia et al. (2023) | * | * | * | * | * | X | 5 |
| Epperla et al. (2025) | * | * | * | * | * | X | 5 |
| Sanber et al. (2023) | * | * | * | * | * | * | 6 |
| Ryan et al. (2023) | * | * | * | * | * | X | 5 |
| Ahmed et al. (2024) | * | * | * | * | * | X | 5 |
| Ahmed et al. (2021) | * | * | * | * | X | X | 4 |
| Xue et al. (2022) | * | * | * | * | * | X | 5 |
| Ayuk et al. (2023) | * | * | * | * | * | X | 5 |
| Karschnia et al. (2022) | * | * | * | * | * | X | 5 |
| Hernández-Tost et al. (2025) | * | * | * | * | * | * | 6 |
| Bennani et al. (2019) | * | * | * | * | * | X | 5 |

# **Supplementary Table 3 – Quality assessment for single-arm retrospective cohort studies (Table S3)**

| Author Name (Year) | Methodological index for non-randomized studies (MINORS) | | | | | | | | Score (Out of 16 ) |
| --- | --- | --- | --- | --- | --- | --- | --- | --- | --- |
|  | A clearly stated aim | Inclusion of consecutive patients | Prospective collection of data | Endpoints appropriate to the aim of the study | Unbiased assessment of the study endpoint | Follow-up period appropriate to the aim of the study | Loss to follow up less than 5% | Prospective calculation of the study size |  |
| Kline et al. (2024) | 2 | 1 | 2 | 2 | 1 | 2 | 2 | 0 | 12 |
| Frigault et al. (2022) | 2 | 2 | 1 | 2 | 2 | 2 | 0 | 0 | 11 |
| Abramson et al. (2020) | 2 | 2 | 2 | 2 | 2 | 2 | 1 | 0 | 13 |
| Liu et al. (2022) | 2 | 2 | 2 | 2 | 1 | 2 | 2 | 0 | 13 |
| Park et al. (2024) | 2 | 1 | 2 | 2 | 1 | 2 | 2 | 1 | 13 |
| Wang et al. (2023) | 2 | 1 | 2 | 2 | 2 | 2 | 1 | 0 | 12 |
| Nayak et al. (2024) | 2 | 1 | 2 | 2 | 1 | 2 | 1 | 0 | 11 |
| Wu et al. (2021) | 2 | 1 | 1 | 2 | 1 | 2 | 1 | 0 | 10 |
| Siddiqi et al. (2021) | 2 | 1 | 2 | 2 | 1 | 2 | 2 | 2 | 14 |
| Park et al. (2023) | 2 | 1 | 2 | 2 | 2 | 2 | 2 | 1 | 14 |
| Ghafouri et al. (2021) | 2 | 1 | 0 | 2 | 1 | 2 | 2 | 0 | 10 |

# **Supplementary Table 4 – Quality assessment for single-arm interventional studies (Table S4)**

| **Study** | **mOS (months)** | **mPFS (months)** |
| --- | --- | --- |
| **Epperla et al. 2023** | **7.6** | **3.3** |
| **Zhou et al. 2024** | **N/A** | **4.72** |
| **Lacan et al. 2023** | **15** | **3** |
| **Alsouqi et al. 2023** | **8.6** | **2.9** |
| **Choquet et al. 2023** | **21.2** | **8.4** |
| **Alcantara et al. 2022** | **N/A** | **10.17** |
| **Zhang et al. 2022** | **9** | **4** |
| **Shumilov et al. 2023** | **7** | **3.6** |
| **Karschnia et al. 2023** | **N/A** | **2** |
| **Wang et al. 2023** | **18.2** | **15.3** |
| **Epperla et al. 2025** | **8.4** | **3.6** |
| **Nayak et al. 2024** | **26.4** | **14.3** |
| **Ghafouri et al. 2021** | **7** | **3** |
| **Sanber et al. 2023** | **N/A** | **12.9** |
| **Ryan et al. 2023** | **N/A** | **11.7** |
| **Ahmed et al. 2021** | **4.3** | **2.8** |
| **Xue et al. 2022** | **N/A** | **16.3** |
| **Ayuk et al. 2023** | **21** | **3.8** |
| **Karschnia et al. 2022** | **7** | **3** |
| **He et al. 2025** | **N/A** | **5.8** |
| **Median of Medians (95% CI)** | **8.6 (7.0-18.2)** | **3.9 (3.15-9.29)** |

# **Supplementary Table 5 – Median survival outcomes in CAR T-Cell therapy for CNS lymphoma (Table S5)**

| **Category** | \| **CAR-T Product / Target** \| \| --- \| | **N (%) of studies** | **Population** | | |
| --- | --- | --- | --- | --- | --- | --- |
|  |  |  | **PCNSL** | **SCNSL** | **Both** |
| **Commercial CAR-T Products** | Axi-cel | 20(51.3%) | 3(15.5%) | 12(60.0%) | 5(25%) |
|  | Tisa-cel | 19(48.7%) | 3(15.8%) | 9(47.4%) | 7(36.8%) |
|  | Liso-cel | 9(23.1%) | 0(0%) | 5(55.6%) | 4(44.4%) |
|  | Brexu-cel | 7(17.9%) | 0(0%) | 3(42.9%) | 4(57.1%) |
|  | Relma-cel | 2(5.1%) | 0(0%) | 0(0%) | 2(100%) |
| **Single Antigen Targeting** | CD19-directed (non-specified product) | 5(12.8%) | 2(40.0%) | 2(40.0%) | 1(20.0%) |
|  | CD20-directed | 2(5.1%) | 0(0%) | 0(0%) | 2(100%) |
|  | CD22-directed | 1(2.6%) | 0(0%) | 0(0%) | 1(100%) |
|  | CD30-directed | 1(2.6%) | 0(0%) | 1(100%) | 0(0%) |
| **Dual Targeting Constructs** | CD19/CD20 | 2(5.1%) | 0(0%) | 1(50.0%) | 1(50.0%) |
|  | CD19/CD22 | 3(7.7%) | 0(0%) | 2(66.7%) | 1(33.3%) |
|  | CD20/CD22 | 1(2.6%) | 0(0%) | 1(100%) | 0(0%) |
| **Other / 3rd Generation** | 1928zT2 cells | 1(2.6%) | 0(0%) | 1(50.0%) | 1(50.0%) |
|  | CD19/CD20/CD22 | 2(5.1%) | 0(0%) | 0(0%) | 1(100%) |
|  | 4-1BB-based CD19/CD20/CD22 | 1(2.6%) | 0(0%) | 1(100%) | 0(0%) |

Prior ASCT was reported in 30 of 39 studies (76.9%), including 345 patients.

# **Supplementary Table 6 – Distribution of CAR T-Cell Products and Target Constructs Stratified by Population in Included Studies (Table S6)**

| **CAR-T Product** | **USA** | **China** | **France** | **Germany** | **Switzerland** | **EU multicenter** | **N/A** | **Total Studies** |
| --- | --- | --- | --- | --- | --- | --- | --- | --- |
| **Axi-cel** | 11 | 3 | 2 | 1 | 1 | 1 | 1 | 20 |
| **Tisa-cel** | 11 | 2 | 3 | 1 | 0 | 1 | 1 | 19 |
| **Liso-cel** | 5 | 0 | 0 | 0 | 0 | 0 | 1 | 9 |
| **Brexu-cel** | 5 | 0 | 0 | 1 | 0 | 1 | 0 | 7 |
| **Relma-cel** | 0 | 2 | 0 | 0 | 0 | 0 | 0 | 2 |
| **CD19-directed** | 2 | 2 | 0 | 1 | 0 | 0 | 0 | 5 |
| **CD20-directed** | 0 | 2 | 0 | 0 | 0 | 0 | 0 | 2 |
| **CD22-directed** | 0 | 1 | 0 | 0 | 0 | 0 | 0 | 1 |
| **CD30-directed** | 0 | 1 | 0 | 0 | 0 | 0 | 0 | 1 |
| **CD19/CD20** | 0 | 2 | 0 | 0 | 0 | 0 | 0 | 2 |
| **CD19/CD22** | 0 | 3 | 0 | 0 | 0 | 0 | 0 | 3 |
| **CD20/CD22** | 0 | 1 | 0 | 0 | 0 | 0 | 0 | 1 |
| **CD19/CD20/CD22** | 1 | 1 | 0 | 0 | 0 | 0 | 0 | 2 |
| **4-1BB CD19/20/22** | 1 | 0 | 0 | 0 | 0 | 0 | 0 | 1 |
| **1928zT2 cells** | 1 | 0 | 0 | 0 | 0 | 0 | 0 | 1 |
| **No. of products used** | 7 | 11 | 2 | 4 | 1 | 3 | 3 | - |

# **Supplementary Table 7 – Cross-Study Frequency and Geographic Distribution of CAR T-Cell Products in CNS Lymphoma Studies (Table S7)**

# **References**

1. Wells G, Shea B, O’Connell D, Peterson J, Welch V, Loso M, et al. Newcastle-Ottawa Scale (NOS) | Ottawa Hospital Research Institute [Internet]. [cited 2026 Jan 11]. Available from: https://ohri.ca/en/who-we-are/core-facilities-and-platforms/ottawa-methods-centre/newcastle-ottawa-scale

2. Slim K, Nini E, Forestier D, Kwiatkowski F, Panis Y, Chipponi J. Methodological index for non-randomized studies (minors): development and validation of a new instrument. ANZ J Surg. 2003 Sep;73(9):712–6. doi:10.1046/j.1445-2197.2003.02748.x PubMed PMID: 12956787.
