# Supplementary figures and images for: Safety and efficacy of CAR T-cell therapy in central nervous system lymphoma: a systematic review and meta-analysis
Source: Front Oncol. 2026 Mar 23;16:1790444. doi: 10.3389/fonc.2026.1790444 (PMC13050687; doi:10.3389/fonc.2026.1790444)

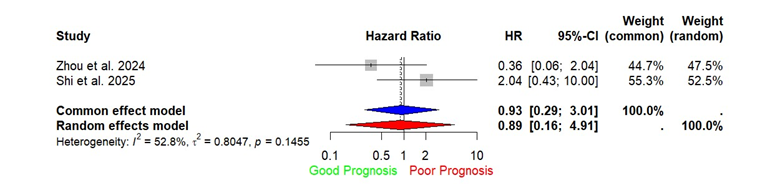

Supplement: Supplementary Figure 1 — Forest plot of pooled hazard ratios (HRs) for progression-free survival (PFS) comparing primary CNS lymphoma (PCNSL) versus secondary CNS lymphoma (SCNSL). Individual study HRs with 95% confidence intervals are shown, along with common-effect and random-effects pooled estimates. Values <1 favor improved prognosis for PCNSL relative to SCNSL. Heterogeneity statistics (I², τ², and p-value) are reported. [file Image1.tif]

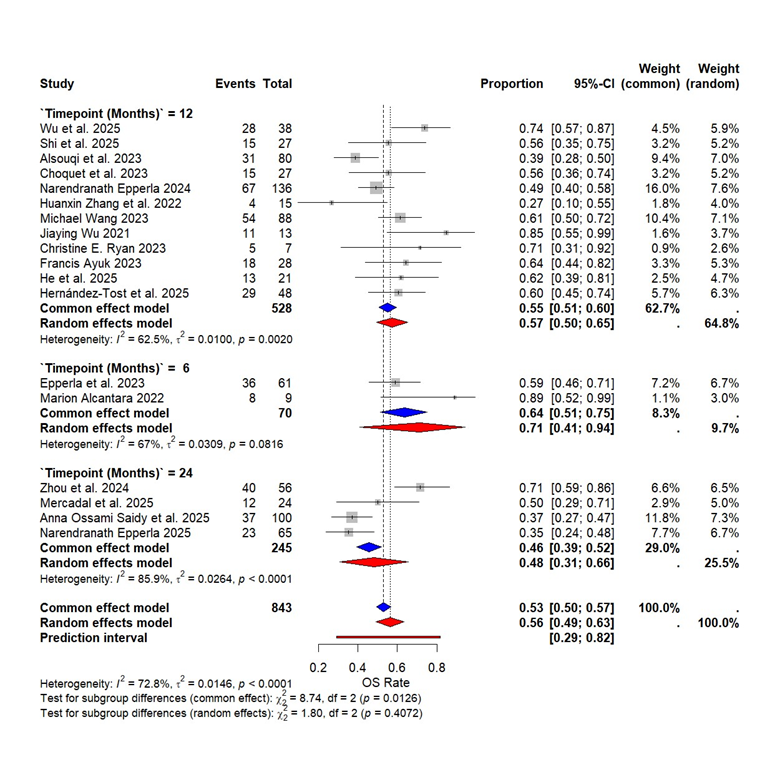

Supplement: Supplementary Figure 2 — Forest plot of pooled overall survival (OS) rates stratified by follow-up time point (6, 12, and 24 months). Individual study proportions with 95% confidence intervals are displayed, with marker size proportional to study weight. Common-effect and random-effects pooled estimates are shown for each subgroup and overall. Heterogeneity statistics (I², τ², and p-values), subgroup comparison tests, and prediction intervals for the random-effects model are reported. [file Image2.tif]

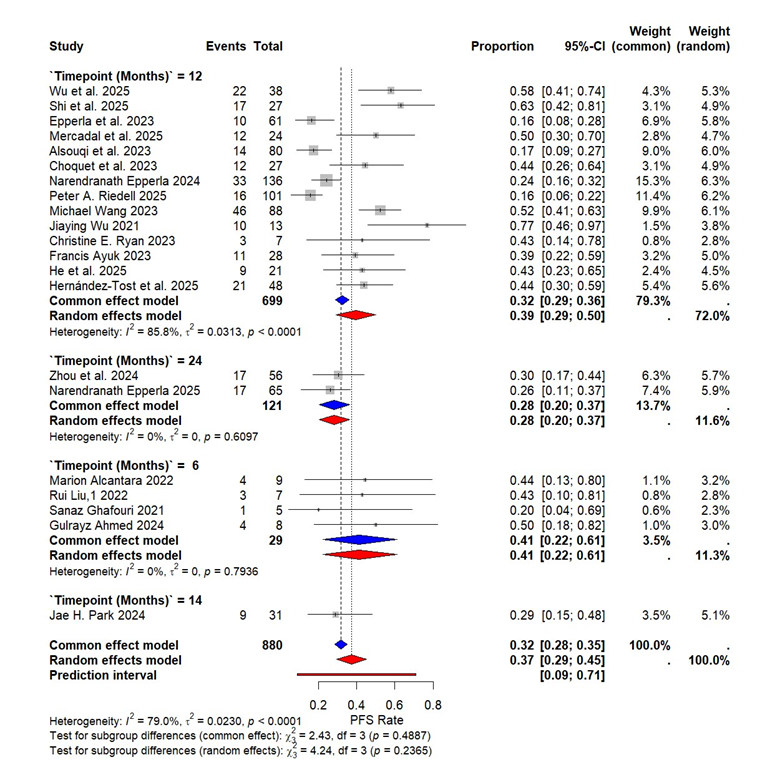

Supplement: Supplementary Figure 3 — Forest plot of pooled progression-free survival (PFS) rates stratified by follow-up time point (3, 6, 9, and 12 months). Individual study proportions with 95% confidence intervals are shown, along with common-effect and random-effects pooled estimates. Overall pooled PFS rates and subgroup heterogeneity statistics (I² and p-values) are presented. Prediction intervals are displayed for random-effects models where applicable. [file Image3.tif]

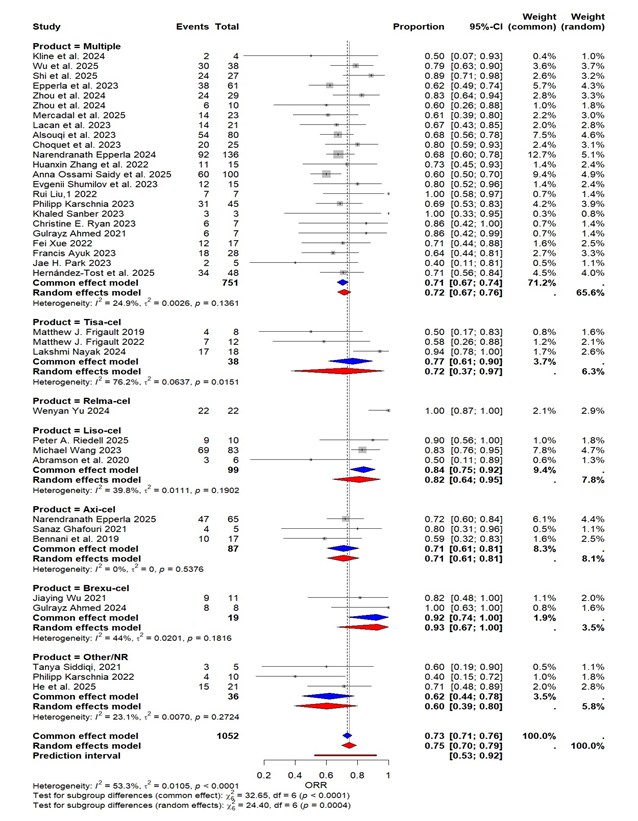

Supplement: Supplementary Figure 4 — Forest plot of pooled objective response rates (ORR) stratified by CAR-T product (multiple products, tisa-cel, liso-cel, Yescarta, axi-cel, brexu-cel, and other/NR). Multiple: Studies including ≥2 CAR-T products (e.g., Axi-cel + Tisa-cel + Liso-cel ± Brexu-cel); Tisa-cel: Axicabtagene ciloleucel only; Liso-cel: Lisocabtagene maraleucel only; Axi-cel: Axicabtagene ciloleucel only; Brexu-cel: Brexucabtagene autoleucel only; Other/NR: CD19-directed (generic/unspecified); 4-1BB–based targeting CD19/CD20/CD22, 1928ζ-CD19 CAR. [file Image4.jpeg]

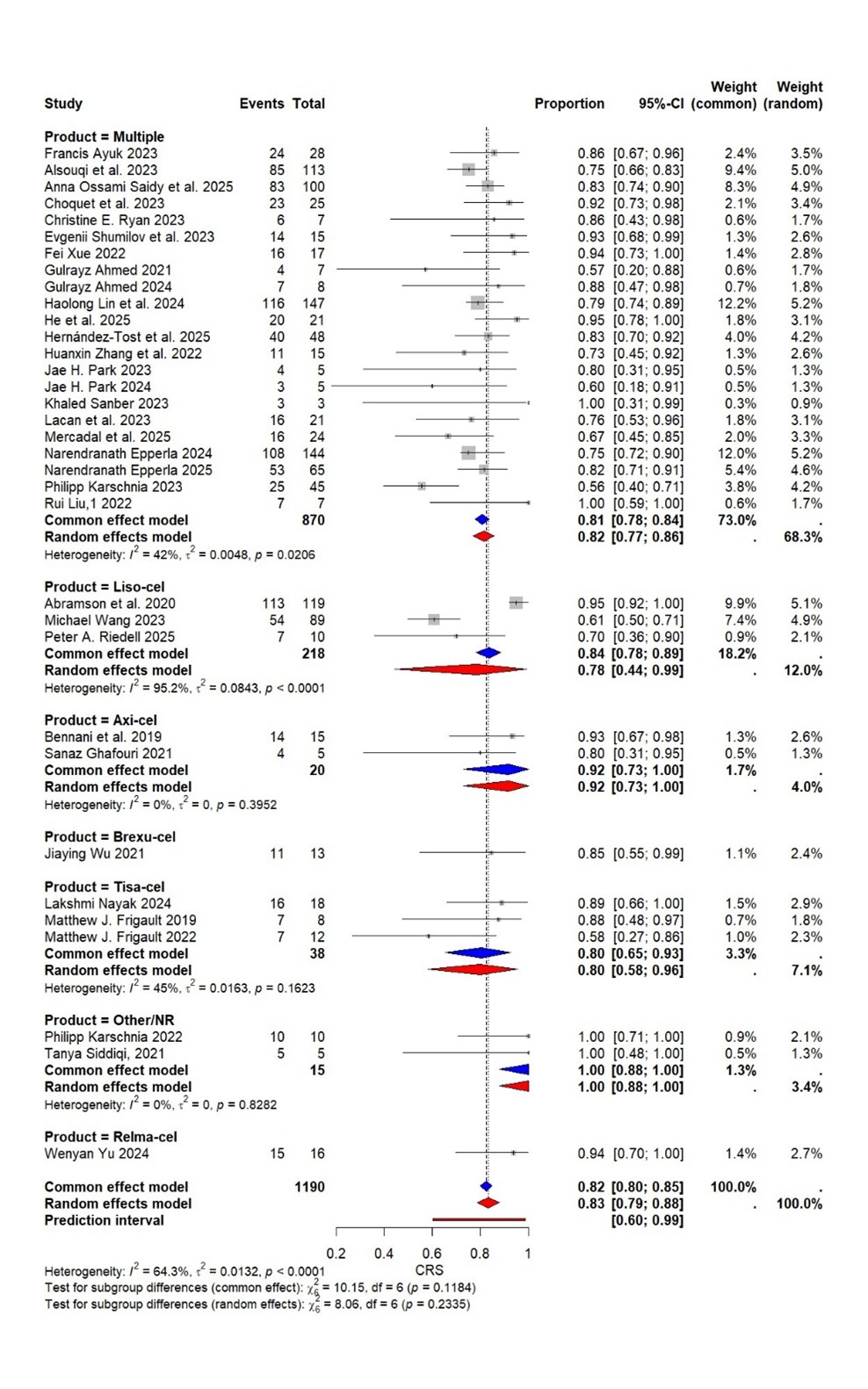

Supplement: Supplementary Figure 5 — Forest plot of pooled cytokine release syndrome (CRS) stratified by CAR-T product (multiple products, tisa-cel, liso-cel, Yescarta, axi-cel, brexu-cel, and other/NR). Multiple: Studies including ≥2 CAR-T products (e.g., Axi-cel + Tisa-cel + Liso-cel ± Brexu-cel); Tisa-cel: Axicabtagene ciloleucel only; Liso-cel: Lisocabtagene maraleucel only; Axi-cel: Axicabtagene ciloleucel only; Brexu-cel: Brexucabtagene autoleucel only; Other/NR: CD19-directed (generic/unspecified); 4-1BB–based targeting CD19/CD20/CD22, 1928ζ-CD19 CAR. [file Image5.jpeg]

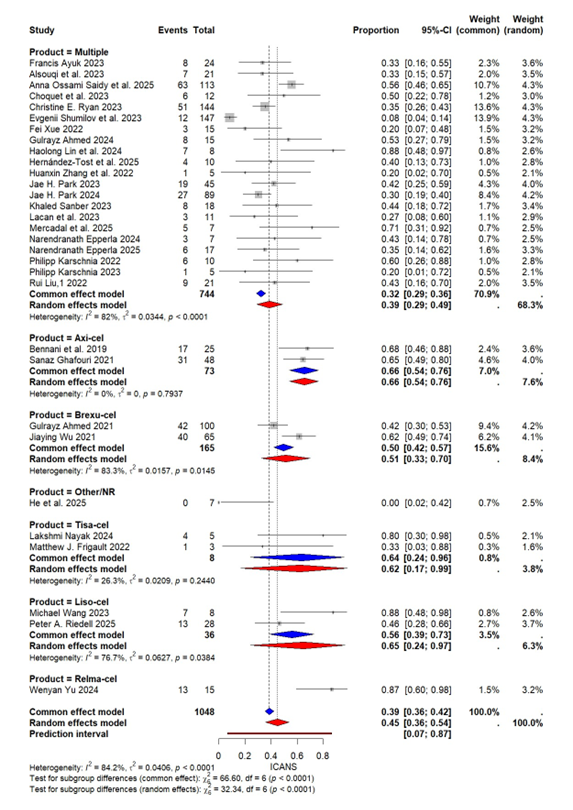

Supplement: Supplementary Figure 6 — Forest plot of pooled Immune Effector Cell–Associated Neurotoxicity Syndrome (ICANS) stratified by CAR-T product (multiple products, tisa-cel, liso-cel, Yescarta, axi-cel, brexu-cel, and other/NR). Multiple: Studies including ≥2 CAR-T products (e.g., Axi-cel + Tisa-cel + Liso-cel ± Brexu-cel); Tisa-cel: Axicabtagene ciloleucel only; Liso-cel: Lisocabtagene maraleucel only; Axi-cel: Axicabtagene ciloleucel only; Brexu-cel: Brexucabtagene autoleucel only; Other/NR: CD19-directed (generic/unspecified); 4-1BB–based targeting CD19/CD20/CD22, 1928ζ-CD19 CAR. [file Image6.tif]
